# “I know a little, but I can’t do more”: a COM-B-based qualitative study of CPAP use and sleep behaviour change among hospitalised patients with COMISA in China

**DOI:** 10.1186/s12890-026-04571-x

**Published:** 2026-09-07

**Authors:** Yuting Wang, Tong Liu, Rong Hu, Weili Zhao, Bixia Zhao

**Affiliations:** School of Nursing, Hengyang Medical School, The Affiliated Nanhua Hospital, University of South China, Hengyang, 421001 Hunan China

**Keywords:** Comorbid insomnia and sleep apnoea (COMISA), Continuous positive airway pressure (CPAP), Cognitive behavioural therapy for insomnia (CBT-I), COM-B model, Theoretical Domains Framework (TDF), Qualitative research, Nursing, Sleep behaviour change

## Abstract

**Background:**

Co-morbid insomnia and sleep apnoea (COMISA) is a highly prevalent condition associated with worse health outcomes and greater treatment complexity than either disorder alone. Patients with COMISA demonstrate lower acceptance and use of continuous positive airway pressure (CPAP) therapy compared with those who have obstructive sleep apnoea (OSA) alone, yet the subjective mechanisms underlying this adherence penalty remain poorly understood. Qualitative research on CPAP use and sleep behaviour change in patients with COMISA is scarce, the COM-B (Capability, Opportunity, Motivation–Behaviour) model has rarely been applied to this population, and the influence of non-Western cultural norms on COMISA treatment engagement has received little attention.

**Methods:**

A descriptive qualitative study guided by the COM-B model and Theoretical Domains Framework (TDF) was conducted at a tertiary sleep medicine centre in Hunan Province, China. Sixteen hospitalised patients with polysomnography-confirmed moderate-to-severe OSA (AHI ≥ 15) and clinically significant insomnia (ISI ≥ 15) were purposively sampled using a 2 × 2 maximum variation matrix based on inpatient CPAP adherence (good vs. poor) and receipt of a brief nurse-led inpatient CBT-I programme (with vs. without structured CBT-I). Individual semi-structured interviews were analysed using directed content analysis, with reporting following the COREQ checklist.

**Results:**

The analysis generated 1,160 reference points across 20 coding nodes, synthesised into three themes and nine sub-themes aligned with the COM-B dimensions. Motivation accounted for the largest share of coded data (589 reference points) and revealed a pattern of “insomnia dominance” in which participants systematically prioritised insomnia over OSA, four patterns of fixed causal attribution that led patients to rule out particular treatments, and an emotion–identity complex linking stigma, age-related identity, and treatment rejection. Within the Opportunity dimension, an “adherence illusion” was identified: some inpatients with good CPAP adherence had not internalised motivation for post-discharge self-management. Culturally specific barriers extended beyond rural CPAP-related death stigma to include urban obligatory banquet drinking norms.

**Conclusions:**

Hospitalised COMISA patients face qualitatively different behavioural barriers from those with OSA alone, requiring COM-B-guided individualised assessment rather than uniform adherence interventions. Nursing practice should incorporate pre-discharge environmental screening, proactive CPAP experience management during the first two to three nights, and culturally sensitive approaches spanning both rural and urban social norms. The Behaviour Change Wheel provides a systematic pathway for translating these behavioural diagnoses into targeted, nurse-led intervention design.

**Supplementary Information:**

The online version contains supplementary material available at https://doi.org/10.1186/s12890-026-04571-x.

## Background

Obstructive sleep apnoea (OSA) and insomnia are the two most prevalent sleep disorders, each affecting a substantial proportion of the adult population. Globally, an estimated 936 million adults aged 30–69 years have OSA, with the largest number living in China [1]; in China, insomnia affects approximately 15% of the general population [2]. These conditions frequently co-occur: approximately 30–50% of patients with OSA report clinically significant insomnia symptoms, and 30–40% of patients with chronic insomnia meet diagnostic criteria for OSA [3, 4]. This overlap, now recognised as co-morbid insomnia and sleep apnoea (COMISA), has emerged as a distinct clinical entity requiring specific diagnostic and treatment approaches [5, 6].

Critically, COMISA is not the simple arithmetic sum of two independent disorders. Evidence from mechanistic studies indicates that insomnia and OSA interact through bi-directional pathways: insomnia-related hyperarousal may elevate the arousal threshold and fragment sleep architecture, while OSA-induced nocturnal awakenings can perpetuate conditioned wakefulness and maladaptive sleep behaviours, creating a self-reinforcing cycle that amplifies the severity of both conditions [7]. The clinical consequences of this interaction are correspondingly more severe. Compared with either disorder alone, COMISA is associated with greater sleep fragmentation, worse daytime functioning, higher rates of depression and anxiety, and elevated cardiovascular morbidity [3]. A population-based analysis of the Sleep Heart Health Study found that COMISA was associated with a 47% increase in all-cause mortality risk over 15 years of follow-up, whereas neither insomnia alone nor OSA alone reached statistical significance for mortality in fully adjusted models [8]. These findings underscore that COMISA constitutes a qualitatively different clinical challenge from OSA or insomnia treated in isolation, demanding integrated assessment and management strategies.

Continuous positive airway pressure (CPAP) is the established first-line treatment for moderate-to-severe OSA, yet long-term adherence remains a persistent clinical challenge. Approximately half of all prescribed patients use CPAP suboptimally, whether through inconsistent nightly use, insufficient hours per night, or outright abandonment [9, 10]. In patients with COMISA, adherence is further compromised: co-morbid insomnia symptoms have been associated with an approximately 30% reduction in initial CPAP acceptance compared with OSA alone, and insomnia-related arousal and sleep fragmentation compound the discomfort of mask-based therapy [11, 12].

Although a substantial quantitative literature has identified predictors of CPAP non-adherence — including disease severity, mask side-effects, self-efficacy, and sociodemographic factors — these studies characterise what predicts adherence without illuminating why patients accept, reject, or abandon treatment. A small but growing body of qualitative research has begun to address this gap by exploring patients’ lived experiences with CPAP [13–15]. However, these studies have focused exclusively on patients with OSA alone or mixed sleep-disordered breathing populations. To date, qualitative research remains limited on how the co-existence of insomnia shapes the subjective experience of CPAP use, including the cognitive appraisals, emotional responses, and competing priorities through which COMISA patients make sense of, engage with, or reject therapy. Consequently, the mechanisms underlying the well-documented adherence penalty associated with comorbid insomnia have received little attention from the patient's perspective.

Emerging evidence suggests that combining CBT-I with CPAP may address the treatment complexities of COMISA more effectively than either therapy alone. A systematic review and meta-analysis found that CBT-I delivered to patients with co-morbid insomnia and sleep apnoea produced clinically meaningful improvements in insomnia severity, and a landmark randomised controlled trial demonstrated that CBT-I prior to CPAP initiation significantly increased subsequent CPAP acceptance and nightly use [5, 12]. These findings position CBT-I as a potentially critical adjunct to CPAP in COMISA management.

However, access to CBT-I remains severely constrained worldwide. The standard protocol requires six to eight weekly sessions delivered by a trained therapist, yet there is a well-documented shortage of qualified CBT-I providers, particularly in low- and middle-income countries and rural regions [16]. In China, where sleep medicine has only recently emerged as a distinct speciality and clinicians formally trained in CBT-I remain scarce, this gap is especially pronounced. Nurse-led models have shown promise in bridging this workforce deficit: nurse-led OSA management has achieved outcomes comparable to physician-led care [17], and nurse-led intensive interventions have improved CPAP adherence [18]. Brief inpatient CBT-I formats have also demonstrated preliminary effectiveness [19]. Yet little is known about how COMISA patients experience these adapted, nurse-delivered interventions or what barriers and facilitators shape their engagement with abbreviated CBT-I programmes.

Understanding why COMISA patients accept, modify, or reject treatment behaviours requires a theoretical framework capable of capturing the full range of determinants — cognitive, environmental, and motivational — that operate simultaneously. The COM-B model (Capability, Opportunity, Motivation–Behaviour) provides such a framework [20]. COM-B posits that behaviour is the product of three interacting components: capability (physical and psychological), opportunity (physical and social), and motivation (reflective and automatic). The Theoretical Domains Framework (TDF), its theoretical elaboration, subdivides these into 14 domains for finer-grained identification of behavioural barriers and facilitators [21].

COM-B was selected over alternative behavioural models — such as the Health Belief Model and Theory of Planned Behaviour — for two principal reasons. First, COM-B explicitly incorporates environmental and structural determinants of behaviour through the Opportunity component, whereas individually focused models tend to emphasise cognitions and intentions while neglecting the physical, social, and institutional conditions under which behaviour occurs. This omission is particularly consequential in COMISA management, where structural barriers such as device accessibility, bedroom infrastructure, and work schedules may be as influential as patient beliefs. Second, COM-B is directly linked to the Behaviour Change Wheel (BCW), a systematic framework that maps behavioural diagnoses onto specific intervention functions and policy categories [20], enabling a seamless translation from research findings to actionable clinical recommendations.

To our knowledge, COM-B has not previously been applied to investigate CPAP adherence or sleep behaviour change in patients with either OSA or COMISA.

Despite the clinical significance of COMISA and the well-documented challenges of managing these co-morbid conditions, several important evidence gaps remain. First, qualitative research specifically examining the experiences, barriers, and facilitators of CPAP use and sleep behaviour change among patients with COMISA is scarce. The small number of existing qualitative studies in this field have focused on OSA-only populations, leaving the distinctive subjective mechanisms through which co-morbid insomnia shapes treatment behaviour underexplored. Second, to our knowledge the COM-B/TDF framework has not been applied to CPAP adherence or sleep behaviour change in either OSA or COMISA populations, despite the framework’s demonstrated utility in other chronic disease contexts. Third, the existing literature has been conducted predominantly in Western, English-speaking settings and has rarely considered how culturally specific norms, such as those surrounding medical device stigma in rural Chinese communities or obligatory social drinking practices in urban professional culture, may influence treatment engagement [22].

This study aimed to address these gaps by exploring the experiences, barriers, and facilitators associated with CPAP use and sleep behaviour change among hospitalised patients with COMISA in China, using the COM-B model and TDF as the guiding theoretical framework. Two research questions were posed: (1) What capability, opportunity, and motivation factors shape hospitalised COMISA patients’ CPAP use and sleep behaviour change? (2) How do these factors differ across patients with varying CPAP adherence and CBT-I exposure?

## Methods

### Study design

A descriptive qualitative study [23] was conducted to explore the experiences, barriers, and facilitators associated with CPAP use and sleep behaviour change among hospitalised patients with COMISA. This design was chosen for its capacity to produce a comprehensive, naturalistic description of the phenomenon under investigation, providing directly applicable evidence for clinical practice [24]. The study was guided by the COM-B model (Capability, Opportunity, Motivation–Behaviour) [20] and its theoretical elaboration, the Theoretical Domains Framework (TDF) [21], which together provided a systematic lens for identifying behavioural barriers and facilitators across three interacting dimensions. Data were analysed using directed content analysis [25], with COM-B/TDF serving as the a priori coding framework while remaining open to emergent themes. This study is reported in accordance with the Consolidated Criteria for Reporting Qualitative Research (COREQ) checklist [26] (Additional file 1).

### Setting and ethics

This study was conducted at the Sleep Medicine Centre of a tertiary hospital affiliated with the University of South China, Hunan Province, China. The centre is situated in a prefecture-level city in a less-developed region and serves a catchment population in which a substantial proportion of patients are from rural areas. It also functions as a regional teaching base for sleep medicine, delivering standardised training in sleep nursing to surrounding hospitals. The centre provides comprehensive inpatient services including polysomnography, CPAP titration, and a one-week structured brief inpatient CBT-I programme. This qualitative study formed part of a larger project on nurse-led cognitive-behavioural interventions for patients with COMISA. Ethical approval was obtained from the Medical Ethics Committee of the University of South China (approval number: 医伦审2025第 (0150) 号). All participants provided written informed consent prior to the interview, were assured of anonymity through alphanumeric coding (P01–P16), and were informed that withdrawal would not affect their clinical care.

### Participants and sampling

Participants were recruited from inpatients at the study site between September 2025 and February 2026. Inclusion criteria were: (a) age ≥ 18 years; (b) PSG-confirmed obstructive sleep apnoea with AHI ≥ 15 events/hour (i.e., moderate-to-severe OSA meeting the indication for CPAP therapy) [27]; (c) clinically significant insomnia with an ISI total score ≥ 15 (moderate or above) [28]; and (d) ability to communicate in Mandarin for 30–60 min. Mandarin is the national language of China and the medium of clinical communication at the study site; no eligible patient was excluded on linguistic grounds. All interviews were conducted by a researcher familiar with the local language environment, and interpreters were not required; direct dialogue was retained to preserve the nuanced expression on which qualitative analysis depends. An age threshold of 18 years was applied because CPAP therapy and CBT-I protocols are established and validated for adults, whereas OSA in those under 18 differs in aetiology, diagnostic criteria, and first-line management, which is typically adenotonsillectomy rather than CPAP; including minors would have introduced a clinically distinct population. Participants were excluded if they had severe cognitive impairment or an unstable psychiatric condition precluding a coherent interview.

A 2 × 2 maximum variation sampling matrix was constructed along two dimensions central to the research questions: inpatient CPAP adherence (good vs. poor) and receipt of the centre’s brief inpatient CBT-I programme (with vs. without structured CBT-I). CPAP adherence was operationally defined for sampling purposes — not as a predictor of long-term use — as follows: “good” denoted continuous use through to discharge with an average of ≥ 4 h per night [29]; “poor” denoted refusal, discontinuation, tapering, or an average of < 4 h per night.

The brief inpatient CBT-I programme was a one-week, nurse-led, structured intervention delivered to all eligible inpatients, though completion varied due to individual preference, financial considerations, or insufficient length of stay. The programme was delivered by nurses holding a national psychological therapist qualification who had received specialised CBT-I training, and comprised six core components: (a) individualised one-to-one cognitive restructuring sessions; (b) a personalised sleep prescription (fixed bedtime–wake schedule); (c) daily sleep diary completed during nursing rounds; (d) one to two group relaxation and mindfulness meditation sessions per week; (e) weekly group psychoeducation on CBT-I principles; and (f) stimulus control implemented through two-hourly overnight nursing rounds, during which nurses identified sleepless patients and guided them to leave the bed for a quiet activity area until drowsiness returned. Nurses also conducted body-surface marking during overnight rounds to independently verify patients’ sleep status, which was cross-referenced with self-reported diary entries the following morning. This abbreviated format was adopted because the standard six-to-eight-week outpatient CBT-I protocol was not feasible in this setting due to patients’ limited financial resources, the predominance of rural patients who could not attend repeated outpatient visits, and the constraints of a one-week average inpatient stay. The programme has been delivered routinely at the centre since its establishment and has been incorporated into the standardised sleep nursing curriculum used for regional training. “With structured CBT-I” was defined as full participation in all six core components of the programme. “Without structured CBT-I” denoted participants who, despite the programme being routinely available to all inpatients, did not receive the full intervention due to personal preference, the out-of-pocket cost of individual sessions, insufficient length of stay, or spending nights away from the ward. These participants may have attended isolated components such as group relaxation sessions or received ad hoc sleep advice during routine nursing care, but did not undergo systematic one-to-one cognitive restructuring or receive a personalised sleep prescription.

This matrix yielded four groups: Group A (good CPAP + with structured CBT-I), Group B (poor CPAP + with structured CBT-I), Group C (good CPAP + without structured CBT-I), and Group D (poor CPAP + without structured CBT-I), with a target of three to four participants per cell. Recruitment continued until informational saturation was observed — operationally defined as the point at which new interviews elaborated existing themes without generating new coding nodes — which occurred after participants 13–14. No participant withdrew or declined to complete the interview; all 16 were retained (retention rate 100%, attrition 0%), ensuring the integrity of the four-cell matrix.

### Data collection

Data were collected through individual, semi-structured, face-to-face interviews conducted by the first author (YTW), a female registered nurse and nursing master’s student who had received training in qualitative research methodology. An interview guide was developed based on the three COM-B dimensions (Capability, Opportunity, Motivation), covering two target behaviours: CPAP use and sleep behaviour change (Additional file 2). The guide comprised four modules: (a) sleep history and diagnostic experience (warm-up); (b) CPAP-related experiences exploring capability, opportunity, and motivation barriers and facilitators; (c) sleep behaviour change experiences along the same three dimensions; and (d) expectations of nurse-led support. Probing questions were used flexibly to follow participants’ narratives. Interviews were conducted between September 2025 and February 2026 in a quiet room on the ward, lasting 40–60 min each. All interviews were audio-recorded with participants’ consent and transcribed verbatim within 48 h. Transcripts were verified against the original recordings by a second researcher (TL). Member checking was not conducted. Field notes documenting non-verbal cues (e.g., sighing, frowning, voice changes) were recorded during and immediately after each interview to supplement the verbal data.

### Data analysis

Data were analysed using directed content analysis [25], a method suited to studies that seek to validate or extend an existing theoretical framework in a specific population. The COM-B model and TDF provided the a priori coding framework: the 14 TDF domains guided the disaggregation of the three parent COM-B components into finer-grained coding nodes, while the analysis remained open to themes that could not be accommodated within the existing structure.

The analytical process proceeded in three phases. First, the first author (YTW) conducted an initial round of coding on the first ten transcripts, which revealed that some codes remained at the parent COM-B level without sufficient TDF differentiation and that the residual category (Other) had absorbed content better captured within COM-B. Based on this review, the coding manual was revised to version 2.0, comprising 20 nodes across four dimensions: Capability (C1–C4), Motivation (M1–M9), Opportunity (O1–O3), and Other (X1–X3, capturing sleep profile, comorbidities, and culturally specific beliefs respectively). Each node was defined with an operational definition, inclusion and exclusion rules, and guidance for distinguishing commonly confused nodes. A new node (X3: culturally specific beliefs) was added to capture phenomena specific to the Chinese cultural context that could not be fully accommodated within the COM-B framework. All 16 transcripts were then re-coded in a new NVivo 14 (Lumivero) project using this revised manual; the original coding was retained as an audit trail.

Second, inter-rater reliability was assessed following the procedure described by Miles and Huberman [30]. A second coder (TL), a nursing master’s student independent of the data collection, was trained using the coding manual v2.0 and a calibration exercise on a practice transcript (P10). She then independently coded four transcripts — one from each sampling group (P08, P13, P05, P02) — using sentence-by-sentence annotation in Word. Percentage agreement was calculated as agreements ÷ (agreements + disagreements) × 100%, with a target of ≥ 85%. Agreement across the four transcripts ranged from 84.8% to 89.2% (mean 86.7%), exceeding the recommended threshold of 85%.

Third, coded data were organised into themes and sub-themes through an iterative process of comparing, contrasting, and clustering nodes within and across the four sampling groups. The corresponding author (BXZ) reviewed all coding outputs and the emerging thematic structure. A reflective journal was maintained throughout, with entries written immediately after coding each transcript (16 entries total), documenting emerging impressions, coding decisions, and the researcher’s positionality as a clinical nurse at the study site (Additional file 3).

### Trustworthiness

Trustworthiness was established according to Lincoln and Guba’s [31] four criteria. Credibility was enhanced through investigator triangulation (coding outputs reviewed by the corresponding author, BXZ), inter-rater reliability testing with an independent second coder (TL), and the inclusion of verbatim quotations to ground all findings in participants’ own words. Transferability was supported through thick description of the study context, detailed participant characteristics, and the use of a maximum variation sampling strategy to capture diverse experiences. Dependability was maintained through a transparent audit trail comprising the iterative development of the coding manual (v1.0 to v2.0), retention of both the initial and final NVivo coding projects, and 16 post-coding reflective journal entries (Additional file 3). Confirmability was addressed through a reflexive statement: the first author (YTW) was a registered nurse undertaking supervised clinical training at the study site as part of her nursing master’s programme, and thus held a dual clinician-researcher role. During her training, she assisted with routine ward nursing care but was not the primary nurse responsible for any participant’s clinical management, did not practise independently, and was not involved in delivering participants’ CPAP titration or CBT-I programme. Participants were informed at recruitment that participation or withdrawal would not affect their clinical care. This positionality afforded insider knowledge of the clinical context and facilitated rapport with participants, while the absence of a direct therapeutic relationship with the behaviours under study reduced the risk of social desirability bias. Nevertheless, the risk of assumptions or premature interpretation remained. These tensions were actively documented in the reflective journal — for instance, instances in which the clinical instinct to correct patients’ misconceptions about CPAP conflicted with the interview principle of non-directive inquiry — and were discussed with the research team during analysis. This study is reported in accordance with the COREQ 32-item checklist [26] (Additional file 1).

During manuscript preparation, an AI-assisted writing tool (Claude, Anthropic) was used to support English-language drafting and editing. The study design, data collection, coding, analysis, and interpretation of all findings were conducted entirely by the human research team. The first author reviewed and verified all AI-assisted output for accuracy and fidelity to the original data.

## Results

**Fig. 1:**
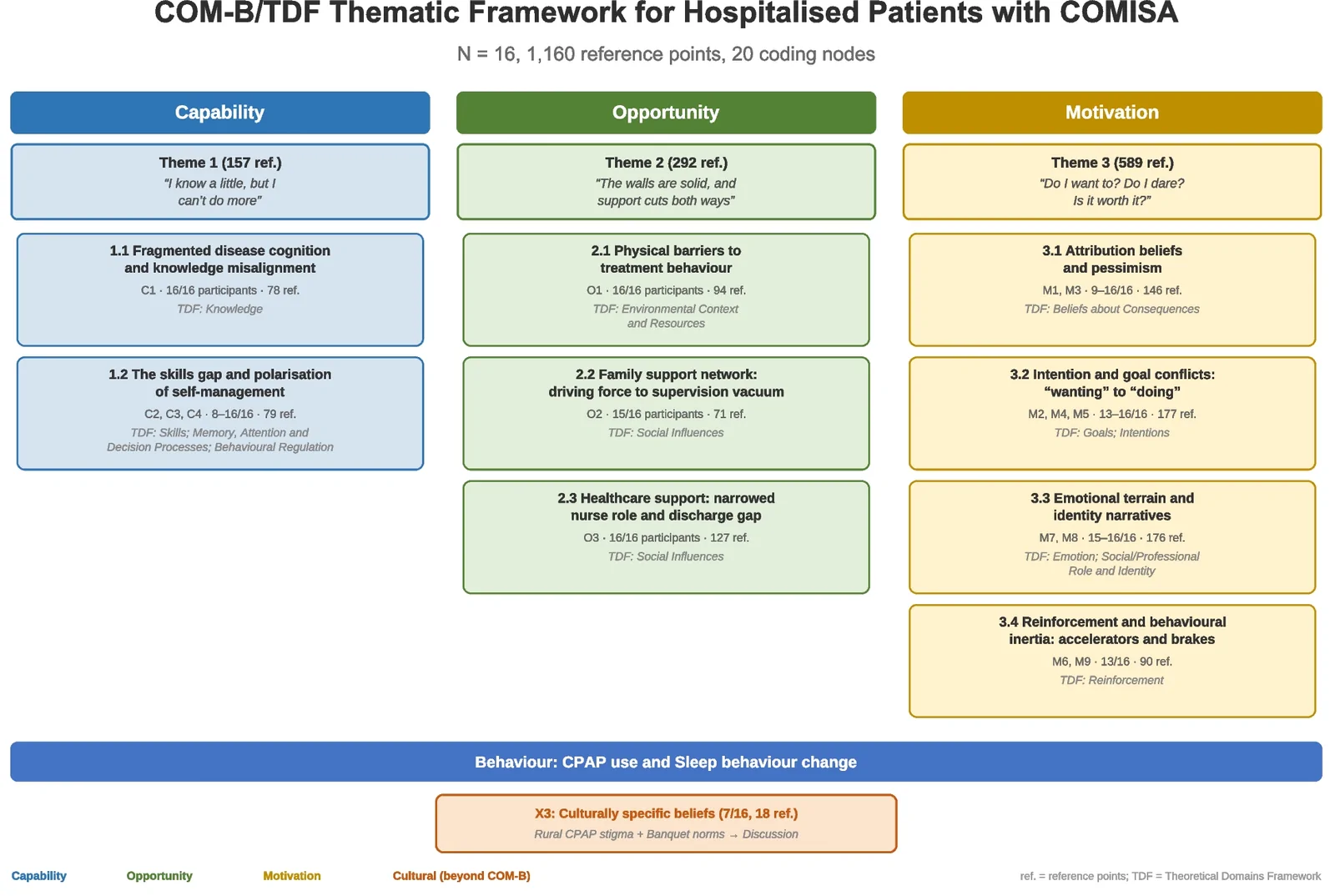
Thematic framework of CPAP use and sleep behaviour change in hospitalised patients with COMISA. Three themes aligned with the COM-B dimensions were identified from directed content analysis of 16 interview transcripts (1,160 reference points). Sub-themes are mapped to corresponding Theoretical Domains Framework (TDF) domains. X3 (culturally specific beliefs) could not be accommodated within the COM-B framework and is discussed separately in the Discussion. Ref. = reference points; participant counts are shown as n/16, with ranges indicating variation across component nodes within multi-node sub-themes

Sixteen hospitalised patients with COMISA were recruited between September 2025 and February 2026. Participants ranged in age from 18 to 70 years (mean 54.4, SD 13.5), with ten males and six females. Educational attainment varied considerably, from no formal education (*n* = 1) to university degree (*n* = 2), with the majority having completed junior high school or senior high school (*n* = 9). Twelve participants were married, three were widowed and living alone, and one was single. All participants had confirmed OSA with AHI ranging from 16.3 to 75.3 events/hour (mean 38.2, SD 17.2); six had moderate and ten had severe OSA. All met the ISI threshold for clinically significant insomnia (ISI range 15–24, mean 18.8, SD 2.9), with 13 classified as moderate and three as severe. Anxiety and depression levels varied widely (HAMA range 5–36, mean 16.2, SD 9.2; HAMD range 5–30, mean 16.4, SD 7.9), reflecting the heterogeneity characteristic of COMISA populations. Detailed participant characteristics are presented in Table 1. Participants were purposively allocated into a 2 × 2 maximum variation matrix based on CPAP tolerance (good vs. poor) and brief inpatient CBT-I experience (with vs. without), yielding four groups of four participants each: Group A (good CPAP adherence + with structured CBT-I, *n* = 4), Group B (poor CPAP adherence + with structured CBT-I, *n* = 4), Group C (good CPAP adherence + without structured CBT-I, *n* = 4), and Group D (poor CPAP adherence + without structured CBT-I, *n* = 4). The four groups differed in demographic profile: Group B was the oldest (mean age 66.2) and included two widowed participants living alone and three from rural backgrounds, while Group C was the youngest (mean age 42.8) and included the youngest participant (P14, aged 18). Interviews lasted 40–60 min each. The directed content analysis generated 1,160 reference points across 20 coding nodes, which were synthesised into three themes and nine sub-themes organised along the COM-B dimensions (Table 2; Fig. 1).

**Table 1:** Participant demographic and clinical characteristics (*N* = 16)

Group	ID	Sex	Age (yr)	Education	Occupation	Marital status	AHI (events/h)	ISI	HAMA	HAMD	CPAP (h/night)	CBT-I
**Group A**	P01	M	62	Senior high school	Retired	Married	17.4	18	11	6	6	With
	P08	M	54	Senior high school	Railway worker	Married	39.8	21	8	7	6	With
	P09	M	64	Senior high school	Retired	Widowed	17.2	19	21	21	6	With
	P10	F	61	Junior high school	Retired	Married	40.2	15	14	19	5	With
**Group B**	P04	F	69	Junior high school	Farmer	Widowed	23.0	17	12	16	—	With
	P07	M	66	Junior high school	Farmer	Married	57.0	16	12	19	1	With
	P11	M	70	Primary school	Retired	Widowed	75.3	18	12	17	4^†^	With
	P13	F	60	Illiterate	Farmer	Married	43.0	16	13	14	—	With
**Group C**	P05	M	43	Senior high school	E-commerce	Married	35.2	15	11	13	5	Without
	P12	M	50	Junior high school	Carpenter	Married	34.5	22	13	16	5	Without
	P14	M	18	Senior high school	Student	Single	26.4	23	34	25	5	Without
	P15	M	60	College	Engineer	Married	61.9	19	5	6	5	Without
**Group D**	P02	F	53	Primary school	Retired	Married	28.1	20	19	19	—	Without
	P03	F	60	Primary school	Retired	Married	40.9	16	10	5	—	Without
	P06	M	42	University	Teacher	Married	55.1	21	36	30	6^‡^	Without
	P16	F	39	University	Teacher	Married	16.3	24	29	30	—	Without
*Total/Mean (SD)*	*N* = *16*	*M:10, F:6*	*54.4 (13.5)*			*M:12, W:3, S:1*	*38.2 (17.2)*	*18.8 (2.9)*	*16.2 (9.2)*	*16.4 (7.9)*	*Good:8, Poor:8*	*With:8, Without:8*

AHI = apnoea–hypopnoea index; ISI = Insomnia Severity Index (moderate 15–21, severe 22–28); HAMA = Hamilton Anxiety Rating Scale; HAMD = Hamilton Depression Rating Scale; CPAP = continuous positive airway pressure; CBT-I = cognitive behavioural therapy for insomnia (With = full participation in all core components of the structured CBT-I programme; Without = did not receive the full programme due to personal preference, out-of-pocket cost, insufficient length of stay, or spending nights away from the ward). Good CPAP adherence = continuous use through discharge with ≥ 4 h/night; Poor = refusal, discontinuation, tapering, or < 4 h/night. — = CPAP refused or not trialled. †P11 initially reached approximately 4 h/night, but use declined from night 4 and was not sustained to discharge; therefore classified as poor adherence. ‡P06 initially used CPAP for approximately 6 h/night, but discontinued after interview and did not sustain use to discharge; therefore classified as poor adherence

**Table 2:** Overview of themes and sub-themes identified through directed content analysis (N = 16, 1,160 reference points)

COM-B dimension	Theme	Sub-theme	Nodes	TDF Domain	n/16	Ref
**Capability**	**Theme 1** *“I know a little, but I can’t do more”*	1.1 Fragmented disease cognition and knowledge misalignment	C1	Knowledge	16	78
		1.2 The skills gap and polarisation of self-management	C2, C3, C4	Skills; Memory, Attention and Decision Processes; Behavioural Regulation	8–16	79
**Opportunity**	**Theme 2** *“The walls are solid, and support cuts both ways”*	2.1 Physical barriers to treatment behaviour	O1	Environmental Context and Resources	16	94
		2.2 The family support network: from driving force to supervision vacuum	O2	Social Influences	15	71
		2.3 Healthcare support: the narrowed nurse role and the inpatient-to-discharge gap	O3	Social Influences	16	127
**Motivation**	**Theme 3** *“Do I want to? Do I dare? Is it worth it?”*	3.1 Attribution beliefs and pessimism	M1, M3	Beliefs about Consequences	9–16	146
		3.2 Intention and goal conflicts: the gap from “wanting” to “doing”	M2, M4, M5	Goals; Intentions	13–16	177
		3.3 Emotional terrain and identity narratives	M7, M8	Emotion; Social/Professional Role and Identity	15–16	176
		3.4 Reinforcement and behavioural inertia: accelerators and brakes	M6, M9	Reinforcement	13	90

Ref. = reference points coded in NVivo 14; n/16 = number of participants (out of 16) whose transcripts contained at least one reference to the sub-theme; ranges indicate variation across component nodes within multi-node sub-themes. Coding nodes correspond to the 20-node coding manual v2.0 (C = Capability, O = Opportunity, M = Motivation). TDF domains follow the standardised nomenclature of Cane et al. (2012). X1 (sleep profile) and X2 (comorbidities) were treated as background characteristics (Table 1); X3 (culturally specific beliefs; 7/16 participants, 18 reference points) is discussed separately in the Discussion

The coded data were synthesised into three overarching themes aligned with the COM-B dimensions: (1) Capability — “I know a little, but I can’t do more” (2 sub-themes, 157 reference points); (2) Opportunity — “The walls are solid, and support cuts both ways” (3 sub-themes, 292 reference points); and (3) Motivation — “Do I want to? Do I dare? Is it worth it?” (4 sub-themes, 589 reference points). The Motivation dimension accounted for the largest proportion of coded data, reflecting the central role of beliefs, emotions, and identity in shaping hospitalised COMISA patients’ treatment behaviours. An overview of themes and sub-themes is presented in Table 2, and the thematic framework is illustrated in Fig. 1.

### Theme 1: Capability

#### Sub-theme 1.1: Fragmented disease cognition and knowledge misalignment (C1; Knowledge; 16/16 participants, 78 reference points)

A universal yet heterogeneous pattern of knowledge deficits was identified across all 16 participants. The most pervasive misconception was the conflation of CPAP with life-support ventilators or oxygen concentrators (e.g., P02, P11). This confusion spanned all educational levels, though its emotional valence differed markedly. Participants with lower educational attainment tended to associate CPAP with terminal illness and death:

“I’ve never heard of it. Isn’t that for seriously ill people? The only people I know who need ventilators are those who are about to die!” (P02, Group D, primary school education)

“All I know is that very sick people use ventilators — you see them on TV, lying in bed nearly dead with a tube for oxygen.” (P11, Group B, primary school education)

In contrast, participants with higher education could distinguish CPAP from oxygen therapy at a conceptual level, yet this rational understanding did not necessarily translate into acceptance:

“I’ve heard of ventilators, but what I know is the oxygen-delivering kind for hypoxia. What you’re describing is probably not the same thing.” (P16, Group D, university education)

Beyond device misconceptions, a systematic underestimation of OSA risk was evident across all four groups. Even participants who had used CPAP for years trivialised the condition, and the Chinese folk belief equating snoring with sound sleep [32] reinforced this dismissal:

“Isn’t snoring perfectly normal? People even say that snoring means you’re sleeping soundly.” (P03, Group D)

This belief reflected not a lack of formal education at the individual level but a broader absence of public health education specific to sleep-disordered breathing, which left even highly educated participants without an accurate understanding of the risks of OSA.

Notably, how participants acquired disease knowledge varied by age and setting. Rural and older participants relied almost exclusively on healthcare professionals and word of mouth, with several expressing a sense of being excluded from medical knowledge:

“I’m from the countryside — I don’t really understand what you doctors talk about.” (P04, Group B, age 69)

By contrast, younger and urban participants actively sought information through digital channels, including one participant who used artificial intelligence tools to verify his treatment plan:

“I search on Baidu or use AI to check whether the treatment plan makes sense.” (P05, Group C, age 43)

This generational digital divide in health information access has direct implications for how nursing education should be tailored — a point elaborated in the Discussion.

#### Sub-theme 1.2: The skills gap and polarisation of self-management (C2, C3, C4; skills, memory, attention and decision processes, behavioural regulation; 8–16 participants per node, 79 reference points)

Participants’ capacity to operate CPAP and manage their own sleep behaviours diverged sharply — from complete self-sufficiency to total dependence — and this polarisation was shaped not only by individual ability but also by the accessibility and consistency of clinical education.

A striking finding was the absence of systematic CPAP training. Across the sample, at least four distinct pathways to acquiring device skills were identified: instruction by nurses during hospitalisation, parameters preset by physicians with no patient education, training provided by the CPAP manufacturer’s after-sales service (P09), and no training at all. Even among participants with good adherence, gaps were evident. P08 (Group A), who persisted with CPAP throughout his hospital stay, had never been taught to operate the device independently:

“I don’t know how! Nobody taught me — they just put it on me every time.” (P08, Group A)

For some, the barrier was not educational but physical. P07, who had limited right-hand function following a previous illness, faced an insurmountable obstacle to independent device operation:

“My right hand doesn’t work properly, so there’s no way I can adjust the machine by myself.” (P07, Group B)

Self-management capacity was equally polarised. At one extreme, two Group C participants (P12, P15) had spontaneously adopted sleep-regulating behaviours before or independent of clinical instruction. P12, a carpenter with no prior CBT-I exposure, described a self-initiated routine that closely mirrored core CBT-I components:

“I’m already doing these things on my own — I get up at six, go for a walk, get some sunshine.” (P12, Group C)

At the other extreme, participants in Groups A and D described difficulties sustaining behaviours without external monitoring. For P01, the removal of hospital-based supervision triggered a predictable decline:

“At first I just forgot to put [the CPAP machine] on, and then I simply stopped bothering.” (P01, Group A)

Meanwhile, P06 (Group D) expressed a need for highly structured external guidance in managing the treatment and sleep behaviours expected of her, reflecting minimal self-regulatory capacity:

“I wish they could just give me a complete plan — tell me exactly what to do at what time.” (P06, Group D)

Within this dimension, skill acquisition and self-management appeared to differ across the groups. Group A participants (P01, P08, P09) had acquired device skills, but through largely passive or external channels: P08 had never been taught to operate the device independently, P09 received training only from the manufacturer’s after-sales service, and P01 lapsed once hospital-based supervision was withdrawn. Group B’s engagement was obstructed by physical limitation (P07). Group C participants (P05, P12, P15) demonstrated strong autonomous self-management independent of clinical instruction, though P14’s compliance was limited to the inpatient setting. Group D participants (P02, P03, P06, P16), having largely rejected treatment, showed minimal engagement with skills or self-regulation. The integrated cross-group analysis across all nine sub-themes is presented in the Cross-group comparison section and Table 3.

**Table 3:** Cross-group comparison of sub-theme patterns across the 2 × 2 sampling matrix

**Sub-theme**	**Group A** *Good CPAP* + *with structured CBT-I*	**Group B** *Poor CPAP* + *with structured CBT-I*	**Group C** *Good CPAP* + *without structured CBT-I*	**Group D** *Poor CPAP* + *without structured CBT-I*
1.1 Fragmented cognition	+ Gaps compensated by positive reinforcement and support	– Gaps compounded by cultural and emotional barriers	+ Active information seeking; P05 uses AI to verify treatment	– Gaps fused with emotional resistance; OSA systematically dismissed
1.2 Skills and self-management	± Skills supported mainly by passive/external channels; independent operation remained limited in some cases	– Obstructed primarily by physical limitations and limited independent device operation	+ Strong autonomous self-management without formal training	– Near-blank engagement; treatment rejected before skills acquired
2.1 Physical barriers	± CPAP discomfort present but accepted; shift-work conflict (P08)	– Rural infrastructure barriers (no outlet, no activities) + body limitations	± Discomfort tolerated; dormitory privacy concern (P14)	– Discomfort amplified by low motivation as “not worth enduring”
2.2 Family support	± Material support present but supervision vacuum post-discharge (P09)	– Highly unequal: widowed/alone (P04, P11) vs limited support	+ Generally positive; P14 parental pressure triggers resistance	– Weakest or absent; P16 zero family references in entire interview
2.3 Healthcare support	± Rich nurse contact via CBT-I but externally driven; discharge gap highest	± Rich nurse contact but cannot sustain without structure post-discharge	± Basic medical advice; nurse role narrowed to “assistant” (P05)	– Minimal engagement; P16 sole need is medical certificate
3.1 Attribution and pessimism	± Positive or neutral attribution; but P09 existential despair coexists	– Cultural fatalism (P02), cumulative hopelessness (P07), age acceptance	+ Pragmatic evaluation; P14 fixed psychological attribution is exception	– Fixed causal attributions: denial (P03), occupational attribution (P16)
3.2 Intention and goal conflicts	+ Positive intention but externally motivated; fragile post-discharge	± Ambivalent: understand rationale but blocked by competing priorities	+ Strong autonomous intention; P15 purchases CPAP proactively	– Negative intention; P16 instrumentalises illness to leave job
3.3 Emotion and identity	± Surface calm but deep negativity (P09 death wish; P10 grief)	– Illness-burdened identity (P07); caregiver self-sacrifice (P13)	+ Positive identity; P15 zero emotional triggers — purely structural barriers	– Highest emotional density; three distinct stigma sources by age/context
3.4 Reinforcement and habit	+ Second-night positive reinforcement cements continued CPAP use	– Reverse reinforcement: P11 “removal-is-relief” blocks re-engagement	± No strong reinforcement pattern; adherence driven by pragmatic choice	– No positive reinforcement reported; cumulative negative experiences

+ = predominantly positive/facilitative; – = predominantly negative/obstructive; ± = mixed or context-dependent. Group profiles: A = externally driven adherence; B = knowing but unable; C = internally anchored; D = not wanting and not doing. CBT-I = cognitive behavioural therapy for insomnia; CPAP = continuous positive airway pressure

### Theme 2: Opportunity

#### Sub-theme 2.1: Physical barriers to treatment behaviour (O1; environmental context and resources; 16/16 participants, 94 reference points)

Physical discomfort with CPAP was near-universal among those who trialled the device, yet the types of discomfort varied widely — airflow pressure, mask leak, dry mouth, noise, claustrophobia, and rubber odour were all reported. Notably, the same type of discomfort was appraised very differently by different participants: P15 (Group C) described the airflow sensation as “acceptable — not a big deal,” whereas P11 (Group B) found the airflow intolerable from the fourth night onward and discontinued use entirely. Because this was an interview study, we cannot exclude objective differences such as variation in prescribed CPAP pressure or individual tolerance. Nevertheless, the marked divergence in response suggests that physical discomfort alone does not determine adherence, and that the psychological appraisal of discomfort, shaped by motivation, identity, and competing priorities, is an important factor.

Beyond device-related discomfort, a more consequential finding was the structural mismatch between standardised treatment recommendations and patients’ real-life environments. Six distinct types of environmental–treatment conflict emerged from the data, which could be classified into two broad categories.

The first category comprised infrastructure-level barriers concentrated among rural participants. Most strikingly, P04 (Group B) revealed that her bedroom lacked an electrical outlet — a fundamental prerequisite for CPAP use that had never been assessed:

“Does the breathing machine need to be plugged in? There’s no power socket next to my bed.” (P04, Group B, age 69, rural) This example also indicated a basic device-education gap: P04’s uncertainty about whether CPAP required electricity suggested that home-use preparation had not fully addressed the practical requirements of device operation.

Rural participants also described an absence of evening activities that could replace prolonged time in bed — a core component of stimulus control therapy:

“What am I supposed to do if I don’t go to sleep? In the countryside, everyone goes to bed when it gets dark. Where am I supposed to take a walk when it’s pitch black outside?” (P13, Group B, age 60, rural)

P13 had completed the structured CBT-I programme and understood the rationale for stimulus control, but the rural environment made it impossible to apply: the barrier lay in the Opportunity dimension rather than in willingness to follow the programme.

The second category comprised work and social-life barriers concentrated among urban participants. P08’s rotating night shifts directly conflicted with the fixed sleep schedule prescribed during hospitalisation:

“The sleep schedule you gave me is fixed, but my work hours aren’t — that’s where the conflict is.” (P08, Group A, railway worker)

Additional conflicts included loss of privacy in shared rooms, which was particularly salient for P14, an 18-year-old student who also faced similar constraints in his school dormitory (“three people in one ward — I don’t want others to see me”); nocturnal work intrusions for P16, a middle-school head teacher whose students’ parents frequently contacted her at night (“parents message or call me late at night”); and a grief-laden home environment for P10, a bereaved mother who had recently lost her adult son (“everywhere at home I see my son’s shadow”). These barriers were not attributable to patient non-compliance but reflected structural conditions beyond individual control.

#### Sub-theme 2.2: The family support network — from driving force to supervision vacuum (O2; Social Influences; 15/16 participants, 71 reference points)

Family members played multiple roles across the treatment trajectory — as the initial discoverers of snoring, as motivators for seeking medical care, as ongoing supervisors of treatment, and, in some cases, as inadvertent obstacles. The data revealed a full spectrum of family involvement, ranging from comprehensive support to complete absence.

At the positive end, P12’s family (Group C) exemplified full-spectrum engagement: they first noticed his worsening temper, confronted him about his unsuccessful self-treatment attempts (“my family told me off and said I should go to the hospital”), and continued to monitor his CPAP use during hospitalisation. P09’s family (Group A) provided material support — his son and daughter-in-law purchased a CPAP device — but day-to-day supervision was limited, as P09 lived alone following his wife’s death.

At the other end of the spectrum, three widowed participants (P04, P09, P11) faced a supervision vacuum after discharge. P11 articulated this starkly:

“I don’t think I can keep it up once I go home — there’s nobody to supervise me. My wife passed away.” (P11, Group B, age 70, widowed)

Family involvement could also prove counterproductive. For P14, the youngest participant in the study, parental pressure to comply with treatment provoked resistance rather than adherence:

“I don’t want to wear it, but my parents will definitely force me to. (Sighs)” (P14, Group C, age 18)

Notably, P16 (Group D) was the only participant whose entire interview contained no reference to family whatsoever — no mention of her husband, children, or any social support. Her cognitive world appeared entirely consumed by work-related distress, leaving family invisible in her problem space.

#### Sub-theme 2.3: Healthcare support — the narrowed nurse role and the inpatient-to-discharge gap (O3; Social Influences; 16/16 participants, 127 reference points)

Healthcare support (O3) was the second most frequently coded node in the study, reflecting the centrality of healthcare interactions in shaping patients’ treatment experiences. Three key findings emerged, each carrying direct implications for nursing practice.

First, patients’ perceptions of the nurse’s professional role were consistently narrowed. Two participants from different groups, educational backgrounds, and age cohorts independently articulated a reductive view of nurses:

“I think nurses are just doctors’ assistants — mainly giving injections and dispensing medication.” (P05, Group C, age 43, senior high school)

“Nurses just follow the doctors’ instructions, don’t they?” (P13, Group B, age 60, illiterate)

These two framings — the nurse as a functional auxiliary and a subordinate executor — both overlooked nurses’ independent expertise in sleep behaviour management, patient education, and psychosocial support. Notably, P05’s reflective journal entry revealed that his framing stemmed from a rational appraisal of professional boundaries, whereas P13’s derived from her lived experience in rural primary care where nurses’ roles were indeed limited to executing medical orders.

Second, despite this narrowed role perception, participants’ actual expectations of nurses were remarkably diverse: behavioural monitoring (P01: “nurses just supervise me”), psychological support (P05 also acknowledged nurses’ role in “emotional counselling during communication”), medication management (P06: “tell me what this medication is and when to take it”), emotional companionship (P10, a bereaved mother: “I wish nurses would chat with me more — even about other things”), group education (P11: “I like it when nurses organise group sessions”), and even non-medical administrative requests (P16, whose sole stated need from the healthcare team was a medical certificate to resign from her role as head teacher). The same “nurse” role was expected to serve entirely different functions in different patients’ minds.

Third, a systematic adherence gap between hospitalisation and discharge emerged as a recurring pattern. During hospitalisation, externally driven compliance functioned well — nurses supervised behaviour, led group activities, and provided moment-to-moment accountability (e.g., P01, P11). However, participants themselves recognised that this structure would not be sustained after discharge:

“Of course I’m willing to keep doing these things, but I just can’t manage it on my own. The atmosphere in the hospital is good — the nurses lead us and we do it together. At home, I simply can’t.” (P11, Group B, age 70, widowed, living alone)

This inpatient-to-discharge gap was particularly evident in Groups A and C, the two groups characterised by good inpatient CPAP adherence. P09 (Group A), who described his approach as “whatever the nurses say, I just do it,” had no strategy for self-management after discharge. P14 (Group C) wore CPAP every night in hospital but explicitly stated that he would discontinue CPAP after discharge. These cases suggest that high inpatient compliance may mask an absence of internalised motivation — an “adherence illusion” that only becomes visible at the point of transition.

### Theme 3: Motivation

#### Sub-theme 3.1: Attribution beliefs and pessimism — “I don’t think this is a big deal” (M1, M3; Beliefs about Consequences; 16/16 and 9/16 participants, 146 reference points)

Beliefs about consequences (M1) was the most frequently coded node in the entire study (133 reference points, 16/16 participants), indicating that how patients understood the causes and consequences of their condition was the single most influential determinant of treatment behaviour.

A pervasive pattern was the subordination of OSA to insomnia — what might be termed “insomnia dominance.” Because insomnia produced immediate, tangible suffering while OSA consequences were perceived as distant and abstract, patients systematically prioritised insomnia and dismissed OSA as inconsequential. P06 articulated this hierarchy with striking clarity:

“Even if snoring is harmful, that’s a problem for the future. But not being able to sleep — I can’t bear the impact of that right now. If I don’t sleep well tonight, I simply cannot function tomorrow.” (P06, Group D, age 42, teacher)

Beyond this general pattern, four patterns of fixed causal attribution were identified, each leading patients to dismiss a particular treatment option: fatalistic attribution (P02: “I disrupted my body clock years ago — the root of illness was set, and the damage is done”), illness denial (P03: “I feel perfectly fine — everyone around me says I don’t even look like an insomniac”), psychological attribution (P14: “All of this is caused by my anxiety and depression — sleep interventions won’t help”), and occupational attribution (P16: “The root of my problem isn’t here — no matter what you do, it’s futile”). Once these attributions became established, all treatment recommendations inconsistent with the patient’s causal model were systematically rejected.

The most clinically resistant form of rejection was rationalised dismissal — acknowledging CPAP’s general efficacy while excluding oneself from its target population:

“Since CPAP has been widely adopted as a medical device, it obviously works. But I don’t think I’m the right person for it.” (P16, Group D, age 39, university education)

This pattern proved more intractable than fear-based (P02) or denial-based (P03) rejection, because it could not be countered with knowledge alone — the patient’s reasoning was internally coherent.

Although M3 (pessimism) appeared in only nine participants (13 reference points), it exhibited remarkable internal diversity, encompassing seven distinct variants: fatalistic resignation (P02: “the damage is done”), cumulative hopelessness (P07: “I’m riddled with illness”), existential weariness (P09), cultural fatalism (P10: “life and death are fated”), age-based acceptance (P11: “at seventy, it makes no difference”), learned helplessness (P14: “I can’t change anything”), and therapeutic nihilism (P16: “it’s all futile”). The most arresting expression came from P09 — a Group A participant with two years of good CPAP adherence:

“Snoring won’t kill me. And if it did, I’d actually be happy — I’d be free.” (P09, Group A, age 64, widowed)

That such profound existential despair coexisted with exemplary treatment compliance underscores a critical point: good adherence does not equal patient wellbeing, and nursing assessment must look beyond behavioural indicators.

#### Sub-theme 3.2: Intention and goal conflicts — the gap from “wanting” to “doing” (M2, M4, M5; Goals, Intentions; 13–16 participants per node, 177 reference points)

Treatment intention (M4) was the single most discriminating node across the four groups. Groups A and C expressed overwhelmingly positive intentions — P05 (Group C) declared “I want to buy the best CPAP machine there is,” while P15 (Group C) had already purchased a device online and planned to buy a second for his office. Groups B and D expressed overwhelmingly negative or ambivalent intentions — P03 (Group D) stated flatly, “I still don’t want to use it — who wears a machine to sleep? I’m perfectly fine; wearing that thing would make me worse.” The most striking split occurred within a single participant: P14 (Group C) complied fully during hospitalisation (“you are helping me with treatment — I should cooperate, not resist”), yet stated explicitly that he would discontinue CPAP after discharge, revealing a fracture between externally motivated inpatient behaviour and internalised intention.

Goal conflicts (M5) emerged in 13 participants and proved far more varied than anticipated. Six distinct conflict types were identified: disease prioritisation (“let me sleep first — that’s the most urgent thing,” P06); work versus treatment (P08’s rotating shifts conflicting with fixed sleep schedules); caregiver self-sacrifice; academic versus sleep demands (P14: “I have to study at night — of course I’ll stay up late”); occupational-social versus health (P15’s alcohol-laden business entertaining); and illness instrumentalisation (P16: “I just want to get sick enough that they’ll take me off the head-teacher role”). Among these, the caregiver pattern was particularly poignant:

“My husband has had a stroke and may end up fully paralysed. Compared to that, what I have isn’t even a real illness — it’s not worth spending money on.” (P13, Group B, age 60, rural, caregiver)

When multiple CBT-I recommendations were blocked by goal conflicts simultaneously, one participant arrived at an unexpected pragmatic solution. P15, a 60-year-old engineer, found that every behavioural recommendation was obstructed by his work and social obligations — reducing alcohol was impossible due to business entertaining norms, fixed wake times conflicted with late-night socialising, and limiting phone use clashed with work communications. Having exhausted all behavioural options, he pivoted to CPAP as the path of least resistance:

“It’s easier to just wear the breathing machine — much less hassle!” (P15, Group C, age 60, engineer)

This pragmatic technology-over-behaviour preference suggests that for patients whose goal conflicts render behavioural change unrealistic, positioning CPAP as the first-line intervention — rather than a fallback after failed behaviour modification — may be a more effective clinical strategy.

#### Sub-theme 3.3: Emotional terrain and identity narratives — “who I am” determines “what I do” (M7, M8; Emotion, Social/Professional Role and Identity; 15/16 and 16/16 participants, 176 reference points)

Emotion (M7) and role and identity (M8) were deeply intertwined in the data, forming an emotion–identity complex in which patients’ reactions to treatment were rooted in their sense of self. Emotion (M7) was the third most coded node (109 reference points, 15/16 participants), with emotional profiles varying markedly across individuals: chronic anxiety (P02), acute terror (P06: “if I don’t sleep I’ll die”), grief (P10), anxious rumination (P14), and anger fused with cognitive replay (P16: “my brain is playing a film on loop”). Notably, P15 (Group C) was the only participant with zero emotional triggers — no anxiety, no fear, no distress — suggesting that for a subset of the hospitalised patients in this study, behavioural obstacles were purely structural and lifestyle-based, rendering emotion-focused interventions irrelevant.

CPAP-related stigma, a prominent pattern within role and identity (M8), arose from three distinct sources that varied systematically by age and social context. Among rural older adults, stigma was rooted in cultural taboos linking ventilators to death and terminal illness (P09: “I’m afraid people will think I’m seriously ill, or that it’s bad luck”). For the youngest participant, stigma stemmed from peer visibility:

“My classmates would see it. How am I supposed to explain? I don’t want to explain.” (P14, Group C, age 18, student)

Among urban professional women, stigma concerned social image (P16: “it doesn’t sound good to talk about”). These three stigma sources — cultural taboo, peer exposure, and professional image — require fundamentally different destigmatisation strategies.

Age-related identity operated in two opposing directions, both leading to CPAP refusal. Younger participants used age as a reason for exemption (P06: “I’m only in my forties”; P16: “I’m still so young — why would I wear a breathing machine?”). Older participants used age as a reason for unworthiness:

“I don’t deserve something this good — it would be wasted on me. At my age, life and death are fated.” (P10, Group A, age 61, bereaved mother)

The identity narratives at both extremes could obstruct treatment. P15’s self-construction as indispensable (“the construction site can’t function without me”) left him no time for behavioural change, while P07’s collapsed identity (“I’m riddled with illness — I can’t do anything any more”) left him no motivation. One participant was too important to stop; the other felt too broken to start.

#### Sub-theme 3.4: Reinforcement and behavioural inertia — accelerators and brakes (M6, M9; Reinforcement; 13/16 participants per node, 90 reference points)

Early treatment experiences functioned as powerful behavioural accelerators or brakes, and the CPAP second-night experience emerged as a potential divergence point between sustained use and early abandonment. Both P08 and P10 (Group A) reported noticeable improvement on their second night of CPAP use — P08 noted “the second night felt better, and I slept better too,” while P10 described sleeping soundly without nocturnal awakenings for the first time. Both continued CPAP use thereafter. No participant in Group D reported any positive reinforcement experience. In Group B, P11’s trajectory illustrated how rapidly positive reinforcement could reverse: the first three nights were tolerable, but from the fourth night onward the experience deteriorated sharply, and a counter-reinforcement belief took hold — what might be termed a “removal-is-relief” response:

“I actually sleep better without the machine — so I stopped wearing it.” (P11, Group B, age 70)

Once this “removal-is-relief” perception formed, it proved resistant to further intervention attempts, suggesting that the first two to three nights represent a critical window for nursing support.

Negative reinforcement also accumulated across treatment modalities. P02’s single failed trial of sleeping pills (“the first time I slept, the second time it didn’t work, so I stopped”) and P16’s discontinuation of psychiatric medication due to side effects (“drowsiness and nausea”) exemplified a pattern in which each unsuccessful attempt reduced willingness to try the next option — a cumulative erosion of therapeutic openness.

At the opposite end of the temporal spectrum, P09 — the only participant with long-term CPAP use (two years) — provided the sole positive example of behavioural automaticity:

“I’m used to it now — it’s nothing special, just normal use. After all, I’ve been using it for so long!” (P09, Group A, age 64)

This suggests that the ultimate goal of adherence support is not to sustain motivation indefinitely, but to help patients cross the threshold from effortful persistence into automatic habit — from “I have to wear it” to “I just do.”

#### Cross-group comparison

The 2 × 2 sampling matrix revealed that neither CPAP tolerance nor CBT-I experience alone determined behavioural outcomes; rather, it was the interaction of capability, opportunity, and motivation factors that differentiated the four groups’ trajectories. A summary of cross-group patterns across all nine sub-themes is presented in Table 3.

Groups A and C both achieved good inpatient CPAP adherence, but through fundamentally different mechanisms. Group A participants (good CPAP + with structured CBT-I) relied heavily on external drivers such as nurse supervision, physician authority, and structured hospital routines to sustain their adherence. Their capability gaps were compensated by institutional support, and early positive reinforcement (the “second-night improvement”) cemented continued use. However, this externally driven compliance carried a high risk of post-discharge non-adherence, as exemplified by P09’s passive obedience and P01’s prior stop-and-restart cycle. Group C participants (good CPAP + without structured CBT-I), by contrast, demonstrated strong autonomous self-management. They actively sought information (P05), spontaneously adopted sleep-regulating behaviours (P12), and made pragmatic cost–benefit decisions (P15). Crucially, these self-management behaviours predated and were independent of the CBT-I programme; P12, who had no prior CBT-I exposure, had already established a routine that closely mirrored core CBT-I components. The pattern therefore reflected individual disposition rather than variation in CBT-I exposure within the group. Their adherence appeared more internally anchored and potentially more durable — though P14’s inpatient-only compliance remained a notable exception.

Groups B and D both exhibited poor CPAP tolerance, but their underlying barriers diverged sharply. Group B (poor CPAP + with structured CBT-I) represented a “knowing but unable” profile: participants understood the rationale for treatment but were blocked by physical limitations (P07), absent infrastructure (P04), caregiver burden (P13), or reverse reinforcement (P11). Their barriers were predominantly structural and environmental — situated in the Opportunity dimension. Group D (poor CPAP + without structured CBT-I) represented a “not wanting and not doing” profile: participants’ rejection was driven by emotional resistance (P02), illness denial (P03), acute anxiety overwhelming all other priorities (P06), or fixed causal attributions (P16). Their barriers were predominantly cognitive and motivational — seated in the Capability and Motivation dimensions.

This distinction carries direct implications for intervention design. Patients who understand the rationale for treatment but are blocked by structural and environmental barriers may benefit most from environmental restructuring and enablement strategies, whereas patients whose barriers are primarily cognitive and motivational require motivational and educational approaches tailored to their specific attribution patterns. This point is elaborated in the Discussion.

## Discussion

This study is, to our knowledge, the first qualitative investigation to apply the COM-B/TDF framework to explore CPAP use and sleep behaviour change among hospitalised patients with COMISA. The directed content analysis of 16 interviews yielded a multidimensional account of the behavioural barriers and facilitators experienced by this population. Three principal findings merit particular attention.

First, a pervasive pattern of “insomnia dominance” was identified in the Motivation dimension: participants systematically prioritised insomnia over OSA because insomnia produced immediate, tangible suffering whereas OSA consequences were perceived as distant and abstract. This attribution hierarchy appears to be underexplored in studies of OSA-only populations and led to the systematic rejection of CPAP in favour of insomnia-focused remedies, suggesting that COMISA requires a fundamentally different motivational approach from OSA alone. Second, a recurring “adherence illusion” emerged in the Opportunity dimension. Participants who demonstrated exemplary CPAP compliance during hospitalisation had not internalised the motivation to continue post-discharge, revealing a structural gap between institutionally supported adherence and autonomous self-management. Third, culturally specific barriers extended beyond the rural CPAP stigma documented in previous Chinese studies to encompass urban professional norms, most notably obligatory banquet drinking culture, indicating that cultural obstacles to sleep behaviour change are not confined to rural or low-education populations.

These findings are discussed below along the three COM-B dimensions, followed by their implications for culturally sensitive, nurse-led intervention design.

### Capability: fragmented knowledge and the digital health literacy divide

The universal yet heterogeneous pattern of disease knowledge deficits identified in this study is broadly consistent with the existing CPAP adherence literature, which has documented patient confusion between therapeutic CPAP and life-support ventilation in OSA-only populations [10, 14] and identified pre-treatment beliefs, including self-efficacy, risk perception, and outcome expectancies, as significant predictors of subsequent adherence [33]. However, the present findings extend this evidence in two important ways. First, in the COMISA context, knowledge deficits operated at two levels simultaneously: participants misunderstood the device (confusing CPAP with oxygen concentrators) and underestimated the disease (dismissing OSA as benign snoring while fixating on insomnia). This dual-layer misconception appears to be underexplored in studies of OSA alone, where the knowledge gap typically centres on a single condition. Second, the emotional valence of the same misconception varied systematically by education level. Lower-educated and rural participants associated CPAP with death and terminal illness, generating fear-based rejection, whereas higher-educated participants could distinguish CPAP from oxygen therapy at a conceptual level yet still rejected it on other grounds. This distinction suggests that patient education for COMISA cannot follow a one-size-fits-all model; rather, it should be stratified by both literacy level and emotional readiness. Trial evidence supports this stratified approach: a brief motivational enhancement education programme combining video, patient-centred interview, and telephone follow-up increased daily CPAP use by two hours and produced a fourfold increase in the proportion of patients meeting adherence thresholds, with treatment self-efficacy identified as a key mediating variable [34].

A particularly noteworthy finding was the generational divide in health information acquisition. Rural and older participants relied almost exclusively on healthcare professionals and word of mouth, while younger and urban participants actively sought information through digital channels. This pattern mirrors the well-documented digital health literacy gap in China, where rural older adults face significantly lower levels of digital literacy and consequently greater health disparities [35, 36]. One participant (P05) went so far as to use an AI chatbot to verify his treatment plan, a behaviour that, to our knowledge, has not been documented in the sleep medicine literature. A recent scoping review identified a growing range of AI applications for chronic disease self-management, including conversational agents that provide personalised health education, symptom monitoring, and decision support, though concerns about digital literacy barriers and the accuracy of AI-generated content remain unresolved [37]. As AI-powered health tools become increasingly accessible, nursing education strategies may need to evolve accordingly, not only correcting misinformation but also guiding patients toward reliable digital resources while remaining alert to AI-generated health misinformation.

### Opportunity: structural barriers, narrowed nurse roles, and the adherence illusion

The existing CPAP adherence literature has focused predominantly on device-related discomfort as the primary physical barrier to use [10]. The present study identified a broader category of structural barriers that extended well beyond the device itself. Six distinct types of environmental-treatment conflict emerged, spanning two categories: infrastructure-level barriers concentrated among rural participants (absence of electrical outlets near the bed, lack of evening activities to replace prolonged time in bed) and work/social-life barriers concentrated among urban participants (rotating shift work, dormitory privacy, nocturnal work communications, grief-laden home environments). These findings resonate with recent evidence that CPAP barriers encompass environmental, regional, and contextual factors that are often overlooked in clinical assessments [38]. This pattern parallels evidence from other underserved populations: among Aboriginal Australians with OSA, long-term CPAP adherence was significantly lower among those residing in remote rather than urban areas, a disparity attributed in part to differences in living and environmental conditions [39]. Critically, these barriers were not attributable to patient non-compliance but reflected structural conditions beyond individual control, suggesting that pre-discharge environmental assessments should become a routine component of CPAP initiation.

A second key finding within the Opportunity dimension was the consistent narrowing of patients’ perceptions of the nurse’s professional role. Two participants from different groups and educational backgrounds independently characterised nurses as “doctors’ assistants” or subordinate executors of medical orders. Both framings overlooked nurses’ independent expertise in sleep behaviour management and psychosocial support. This stands in marked contrast to the international evidence demonstrating that nurse-led and primary care-based OSA management achieves outcomes comparable to specialist-led care [17, 40, 41], and that nurse-led intensive interventions can significantly improve CPAP adherence [18]. The gap between what nurses can do and what patients believe nurses do represents an untapped opportunity for role expansion in COMISA care. This gap may be particularly pronounced in China, where the nursing career ladder has historically been organised within a management-oriented structure with limited pathways for clinical specialisation, and where the development of advanced practice nursing roles remains at an early stage compared with international counterparts [42].

Third, a systematic adherence gap between hospitalisation and discharge emerged as a recurring pattern. During hospitalisation, externally driven compliance functioned well through nurse supervision, structured activities, and moment-to-moment accountability. However, participants themselves recognised that this structure would not be sustained after discharge. Notably, this gap was evident among inpatients who demonstrated good CPAP adherence, suggesting an “adherence illusion” in which observable adherence may mask an absence of internalised motivation. This pattern is not well characterised in the COMISA literature, though it aligns with the broader observation that early CPAP adherence patterns tend to be established within the first week and predict long-term use, with objective monitoring data suggesting that adherence measured as early as three days after initiation is predictive of use patterns at one month [9, 43]. The clinical implication is that nursing assessment should evaluate not only whether patients are using CPAP but why they are using it, distinguishing between externally motivated compliance and autonomous self-management before discharge. The finding that three widowed participants who lived alone faced a complete supervision vacuum upon discharge is consistent with evidence that patients living alone use CPAP significantly fewer hours per night than those living with a partner, and that collaborative spousal involvement during the first week of treatment predicts higher adherence at three months [44]. For COMISA patients without available family support, alternative monitoring structures such as community nurse follow-up or peer support may be essential to prevent post-discharge non-adherence.

Importantly, receipt of the structured CBT-I programme did not necessarily translate into autonomous implementation of CBT-I recommendations. In this sample, P09 remained dependent on external support rather than developing an independent self-management strategy, P11 discontinued CPAP despite understanding the treatment rationale, and P13 was unable to implement stimulus control because her rural environment offered no feasible evening activity after dark. Conversely, some participants without structured CBT-I demonstrated self-management behaviours that predated clinical instruction. These patterns suggest that structured CBT-I exposure in this cross-sectional sample should be interpreted as a contextual dimension rather than as a direct indicator of behaviour change.

### Motivation: insomnia dominance, fixed causal attributions, and the emotion–identity complex

The Motivation dimension accounted for over half of all coded data (589 of 1,160 reference points), confirming its centrality in COMISA treatment behaviour. The most consequential finding was a pattern of “insomnia dominance” in which participants systematically subordinated OSA to insomnia because insomnia produced immediate suffering whereas OSA consequences were perceived as distant and abstract. This hierarchy has received little attention in the qualitative COMISA literature, though quantitative evidence suggests that co-morbid insomnia symptoms are associated with approximately a 30% reduction in initial CPAP acceptance compared to OSA alone [12]. Longitudinal evidence informed by Leventhal’s Common Sense Model has further shown that patients’ illness representations, including perceived consequences and emotional responses to the diagnosis, independently predict PAP adherence over six months, with self-efficacy mediating the relationship between emotional threat appraisal and device use [45]. The present study adds a qualitative mechanism to this statistical observation: patients do not merely tolerate CPAP less well when insomnia is present; rather, they construct a causal model in which insomnia is the “real” problem and OSA is peripheral, leading to the systematic rejection of any intervention that does not directly address sleeplessness. Four patterns of fixed causal attribution were identified, each leading patients to dismiss a particular treatment option. Crucially, the most clinically resistant form was rationalised dismissal, in which the patient acknowledged CPAP’s general efficacy while excluding themselves from its target population through internally coherent reasoning that could not be countered with knowledge alone. This observation is consistent with trial evidence showing that motivational enhancement, which operates within patients’ existing belief systems rather than directly challenging them, can increase nightly CPAP use by approximately 1.5 h compared with standard education [46], and suggests that for patients with fixed causal attributions, approaches grounded in motivational interviewing may be more appropriate than didactic correction [47].

Early reinforcement experiences proved to be powerful behavioural accelerators or brakes. Participants who reported noticeable improvement on their second night of CPAP use continued therapy, whereas no participant in the poor-adherence groups reported any positive early experience. This aligns with evidence that CPAP adherence patterns are established within the first days of treatment and predict long-term use [9, 48]. Of particular concern was the formation of a “removal-is-relief” response, in which discontinuing CPAP was experienced as rewarding. Once this counter-reinforcement belief formed, it proved resistant to subsequent intervention, suggesting that the first two to three nights represent a critical window for proactive nursing support including expectation management, mask comfort optimisation, and scheduled check-ins.

An unexpected finding was one participant’s pragmatic pivot from behavioural change to technological solution. When every CBT-I recommendation was blocked by occupational and social obligations, he chose CPAP as the path of least resistance. This challenges the prevailing CBT-I-first sequencing recommended for COMISA [5] and suggests that for patients whose goal conflicts render behavioural change unrealistic, positioning CPAP as the first-line intervention may be more effective than insisting on behaviour modification that is unlikely to succeed.

Finally, the interplay between emotion and identity formed a distinctive "emotion-identity complex" that shaped treatment responses. This accords with a recent synthesis of the psychological determinants of CPAP adherence, which positions device-related anxiety, perceived stigma, and psychological comorbidity as factors operating across the predisposing, precipitating, and perpetuating stages of treatment [49]. However, one participant exhibited zero emotional triggers while maintaining good CPAP adherence, with barriers that were purely structural and lifestyle-based. More broadly, the contrast between autonomously managed adherence and externally driven compliance resonates with prospective evidence that active coping strategies, particularly planful problem solving, and higher self-efficacy are significant predictors of sustained CPAP use, whereas passive coping styles are associated with early non-adherence [50]. This finding cautions against assuming that all COMISA patients require emotion-focused interventions and supports the case for COM-B-guided individualised assessment to identify which dimension is the primary driver for each patient.

### Culturally specific barriers beyond the COM-B framework

A distinctive contribution of this study is the identification of culturally embedded barriers to CPAP use and sleep behaviour change that cannot be fully captured within the standard COM-B framework. Seven participants across both rural and urban settings triggered the culturally specific beliefs node (X3), revealing two dimensions of cultural influence that have received little attention in the international sleep literature.

The first dimension was a collective CPAP stigma rooted in rural Chinese folk beliefs. Six participants independently associated CPAP with death, terminal illness, and supernatural misfortune, using strikingly consistent language: “cursing oneself,” “bad luck,” and “only dying people wear ventilators.” This stigma was not an individual misconception but a community-level norm, evidenced by participants’ use of collective pronouns (“in our village,” “everyone thinks”) and their selective concealment of CPAP use when visiting relatives. While CPAP-related embarrassment and self-consciousness have been reported in Western populations, where stigma typically centres on body image, perceived unattractiveness to bed partners, and social awkwardness associated with device visibility [22, 38, 51], the mechanism identified in the present study differs substantially. In the present study, stigma was anchored in culturally specific death taboos, where the visual resemblance between CPAP masks and life-support equipment triggered associations with imminent mortality and spiritual contamination. This distinction carries practical implications: destigmatisation strategies effective in Western contexts (e.g., normalising CPAP as a wellness device) may be insufficient in Chinese rural settings, where the underlying fear is not social judgement but symbolic pollution. Ethnographic research on chronic disease stigma in rural China has demonstrated that stigmatisation operates simultaneously at the internalised, interpersonal, and institutional levels within a specific sociocultural context [52], a multi-layered structure that resonates with the findings of the present study, where CPAP stigma was expressed through individual concealment, family-level selective disclosure, and community-level normative exclusion. Ethnographic and qualitative research on health-seeking behaviour among rural older adults in China has documented a broader pattern in which illness is defined in terms of functional incapacity rather than clinical diagnosis, medical advice is selectively followed, and treatment-seeking is deferred until symptoms become unbearable [53], a pattern that may compound CPAP stigma by reinforcing the belief that asymptomatic conditions such as OSA do not warrant device-based intervention.

The second dimension extended the scope of X3 beyond rural populations. One urban professional participant described obligatory banquet drinking as an insurmountable barrier to reducing alcohol intake, a core CBT-I recommendation. The social norm of “not drinking when toasted means disrespecting the other person” represents a culturally specific form of peer pressure with no direct equivalent in Western sleep literature. Qualitative evidence from rural China has similarly documented that heavy drinking functions as a bonding mechanism through which interpersonal respect is expressed and group identity maintained, with the cultural proverb “true friends empty the bottom” illustrating the social obligation to consume alcohol regardless of personal health considerations [54]. This finding suggests that cultural barriers to sleep behaviour change are not confined to rural, low-education populations but also operate through urban professional social norms. Nursing assessments for COMISA patients in China should therefore incorporate culturally sensitive screening across both rural and urban contexts, and for patients like this one, a harm-reduction approach (e.g., maintaining CPAP use on nights after heavy drinking rather than insisting on abstinence) may be more realistic than prescriptive behavioural rules.

The coding boundary decisions for X3 also warrant brief discussion as a methodological point. We restricted X3 to collectively held cultural norms and excluded individual misconceptions shaped by media exposure (e.g., one participant’s association of CPAP with televised ICU scenes was coded as a knowledge deficit rather than a cultural belief). This distinction between individual cognition and collective norm proved analytically productive and may be useful for future qualitative studies applying behavioural frameworks in culturally diverse populations.

### Clinical implications: mapping barriers to the behaviour change wheel

**Fig. 2:**
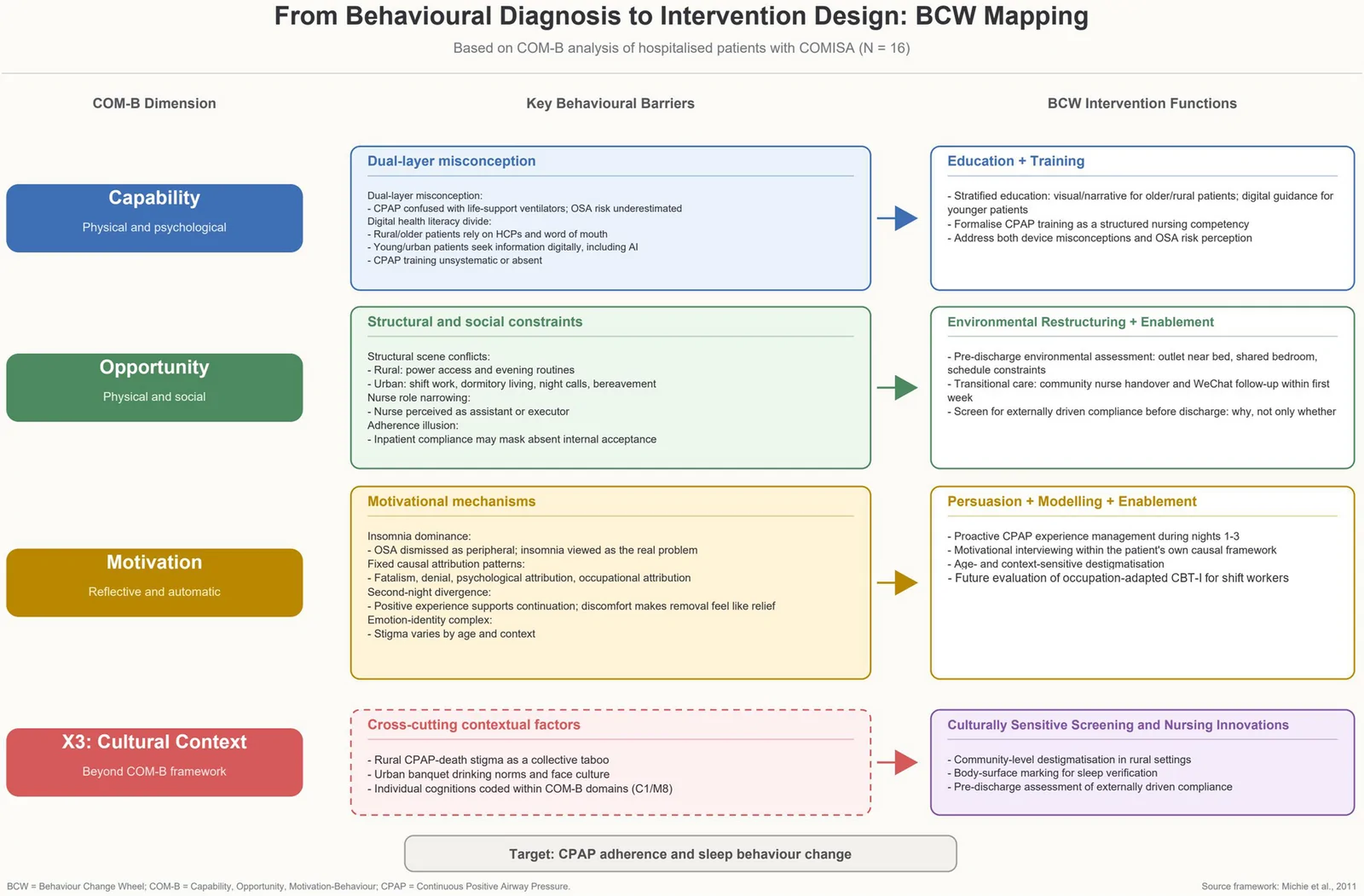
Behavioural barriers and intervention functions in hospitalised patients with COMISA

A key advantage of the COM-B framework is its direct linkage to the Behaviour Change Wheel (BCW), which maps behavioural diagnoses onto specific intervention functions [20]. The present findings suggest that COMISA management requires interventions operating across all three COM-B dimensions simultaneously, rather than targeting any single dimension in isolation (Fig. 2).

For Capability deficits, the indicated BCW functions are Education and Training. The generational divide in health information access identified in this study calls for stratified patient education: visual, narrative-based materials and face-to-face explanations for older and rural patients, complemented by curated digital resources and guidance on evaluating online health information for younger, digitally literate patients. CPAP operational training should be formalised as a structured nursing competency rather than left to ad hoc instruction or device manufacturers.

For Opportunity barriers, the indicated functions are Environmental Restructuring and Enablement. The six structural scene conflicts identified in this study highlight the need for pre-discharge environmental assessments as a routine nursing intervention. Practical questions such as “Is there an electrical outlet near your bed?” and “Do you share a bedroom?” should be incorporated into CPAP initiation protocols. For the inpatient-to-discharge adherence gap, transitional care strategies are essential. These could include structured handover to community nurses, scheduled telephone or WeChat follow-ups within the first week, and early identification of patients relying on externally driven compliance who are at high risk of post-discharge non-adherence. Meta-analytic evidence supports the efficacy of telemonitoring in improving CPAP adherence through timely clinician feedback and problem-solving during the critical early treatment period [55], and such approaches may be particularly valuable for the rural patients in this study who cannot readily access outpatient follow-up.

For Motivation barriers, the indicated functions are Persuasion, Modelling, and Enablement. The finding that early reinforcement experiences function as a divergence point between sustained use and abandonment supports proactive CPAP experience management during the first two to three nights, including expectation setting, scheduled comfort checks, and timely pressure adjustments. For patients with fixed causal attributions, motivational interviewing techniques that work within the patient’s existing causal framework, rather than directly challenging it, may be more productive than standard health education [47].

Several specific nursing practice innovations emerge from this study. First, the overnight body-surface marking technique used at the study site, in which nurses mark a small circle on patients’ skin during two-hourly rounds to independently verify sleep status, represents a low-cost, nurse-led objective sleep assessment method. By cross-referencing these markings with patients’ self-reported sleep diaries the following morning, clinicians obtained a more accurate picture of actual sleep than self-report alone could provide. This approach requires no additional equipment, integrates naturally into existing nursing rounds, and could be readily adopted in resource-limited settings where actigraphy is unavailable. Second, the finding that standardised CBT-I sleep schedules conflicted directly with rotating shift work suggests the need for flexible, occupation-adapted sleep prescriptions. Cognitive behavioural therapy for insomnia has been adapted for shift workers with chronic insomnia [56], including a recent trial among shift-working nurses [57], providing preliminary support for occupation-adapted approaches. Future research should examine whether rotating-compatible sleep windows can preserve the core principles of CBT-I while accommodating occupational demands, and whether appropriately trained nurses can safely and effectively support the delivery and individualisation of such adapted approaches.

Left column: COM-B dimensions identified through directed content analysis. Centre column: key behavioural barriers from the present study. Right column: corresponding BCW intervention functions and proposed clinical and research implications. X3 (culturally specific beliefs) is presented separately as it extends beyond the COM-B framework. BCW = Behaviour Change Wheel [Michie et al. 2011]; CPAP = continuous positive airway pressure; CBT-I = cognitive behavioural therapy for insomnia.

### Limitations

Several limitations should be acknowledged. First, this was a single-centre study conducted at one tertiary sleep medicine centre in Hunan Province, which may limit the transferability of findings to other regions of China or other healthcare systems. Moreover, all participants were hospitalised inpatients, and certain findings, particularly those related to ward privacy, overnight nursing rounds, and the inpatient-to-discharge transition, are specific to the hospital setting and may not apply directly to outpatient or community-based COMISA populations. Second, the cross-sectional design captured participants’ experiences at a single time point during hospitalisation and cannot track how behavioural barriers and facilitators evolve over the longer-term post-discharge trajectory. Longitudinal qualitative follow-up studies are needed to examine whether patterns such as the “adherence illusion” and “removal-is-relief” response persist, resolve, or transform over time. Third, the brief inpatient CBT-I programme delivered at the study site was a one-week structured intervention rather than the standard six-to-eight-week outpatient protocol. While this limits direct comparability with studies using standard-duration CBT-I, the abbreviated format was a pragmatic adaptation to the socioeconomic realities of a less-developed region where most patients cannot afford or access prolonged outpatient treatment. Recent evidence supports the effectiveness of such abbreviated inpatient formats [19], and the model has been successfully integrated into the centre’s regional training programme, suggesting potential for replication in other resource-limited settings across China. Fourth, although the structured CBT-I programme was offered to all inpatients, participants’ reasons for not completing it were heterogeneous, and the with/without classification reflects a gradient of exposure rather than a clean dichotomy. Additionally, as a cross-sectional interview study rather than an intervention trial, we did not systematically assess whether participants who completed the programme subsequently followed its recommendations in practice. Fifth, a declining trend in reference points was observed across the coding sequence (mean 83 per transcript for participants 1–7 versus 66 for participants 8–16), which may reflect coder maturation. This potential effect was monitored through inter-rater reliability testing using transcripts sampled across the full coding timeline. Finally, the first author’s clinical role at the study site and the sole interviewer introduced the possibility of social desirability bias in participants’ responses and interpretive bias in coding. This positionality was actively managed through reflexive journaling after each interview and coding session, regular discussion with the research team, and independent coding verification.

## Conclusions

This study provides a qualitative account of CPAP use and sleep behaviour change among hospitalised patients with COMISA, applying the COM-B/TDF framework to reveal a multidimensional landscape of behavioural barriers and facilitators that differs substantively from that reported in OSA-only populations. Three findings carry particular significance for nursing practice. First, the pervasive pattern of “insomnia dominance” — in which patients subordinate OSA to insomnia because of its immediate, tangible suffering — means that COMISA management cannot simply replicate OSA adherence strategies; it requires motivational approaches that acknowledge and work within patients’ own illness hierarchies. Second, the “adherence illusion” — in which exemplary inpatient compliance masks an absence of internalised motivation — calls for nursing assessment that evaluates not only whether patients use CPAP but why, with particular attention to those transitioning from institutional to home-based care. Third, culturally specific barriers to treatment operate across both rural and urban settings, necessitating culturally sensitive screening that extends beyond the rural stigma documented in previous Chinese studies to encompass urban professional social norms.

The COM-B framework proved well suited to capturing the simultaneous interplay of cognitive, environmental, and motivational factors in COMISA, and its direct linkage to the Behaviour Change Wheel offers a systematic pathway for developing targeted, nurse-led interventions. Future research should test the effectiveness of COM-B-informed intervention packages for COMISA patients and examine whether the behavioural patterns identified here — particularly insomnia dominance and the adherence illusion — persist, resolve, or transform across the post-discharge trajectory. A further direction concerns the youngest participant in this sample, who displayed a striking divergence between outward compliance in hospital and privately held rejection of treatment. As this observation rests on one participant, it cannot support specific clinical recommendations, but it identifies a question worth dedicated study: how young adults with COMISA experience treatment, and what nursing communication approaches best support their engagement.

## Supplementary Information

Additional file 1. COREQ checklist. Completed Consolidated Criteria for Reporting Qualitative Researchchecklist for this study.

Additional file 2. Interview guide. Semi-structured interview guide developed according to the COM-B dimensions and the two target behaviours: CPAP use and sleep behaviour change.

Additional file 3. Reflective journal excerpts. Excerpts documenting reflexivity, coding decisions, and analytic reflections during data analysis.

## Data Availability

The qualitative datasets generated during this study are not publicly available due to the sensitive and potentially identifiable nature of interview transcripts, but are available from the corresponding author upon reasonable request.

## Abbreviations

AHI: Apnoea–Hypopnoea Index
AI: Artificial Intelligence
BCW: Behaviour Change Wheel
CBT-I: Cognitive Behavioural Therapy for Insomnia
COM-B: Capability, Opportunity, Motivation–Behaviour
COMISA: Co-morbid Insomnia and Sleep Apnoea
COREQ: Consolidated Criteria for Reporting Qualitative Research
CPAP: Continuous Positive Airway Pressure
HAMA: Hamilton Anxiety Rating Scale
HAMD: Hamilton Depression Rating Scale
ISI: Insomnia Severity Index
OSA: Obstructive Sleep Apnoea
PSG: Polysomnography
TDF: Theoretical Domains Framework
