# Supplementary material for: “I know a little, but I can’t do more”: a COM-B-based qualitative study of CPAP use and sleep behaviour change among hospitalised patients with COMISA in China
Source: BMC Pulm Med. 2026 Sep 7;26:429. doi: 10.1186/s12890-026-04571-x (PMC13615572; doi:10.1186/s12890-026-04571-x)
Supplement: Supplementary file 1 [file 12890_2026_4571_MOESM1_ESM.docx]

**Additional File 1**

**Consolidated Criteria for Reporting Qualitative Research (COREQ): 32-item checklist**

*Tong A, Sainsbury P, Craig J. Consolidated criteria for reporting qualitative research (COREQ): a 32-item checklist for interviews and focus groups. International Journal for Quality in Health Care. 2007;19(6):349–357.*

| **No.** | **Item** | **Guide questions/description** | **Reported in this manuscript** |
| --- | --- | --- | --- |
| ***Domain 1: Research team and reflexivity*** | | | |
| **Personal characteristics** | | | |
| 1 | Interviewer/facilitator | *Which author/s conducted the interview or focus group?* | Methods, Data collection: The first author (YTW) conducted all interviews. |
| 2 | Credentials | *What were the researcher’s credentials?* | Methods, Data collection: Registered nurse and nursing master’s student. |
| 3 | Occupation | *What was their occupation at the time of the study?* | Methods, Trustworthiness: Clinical nurse at the study site. |
| 4 | Gender | *Was the researcher male or female?* | Methods, Data collection: The interviewer (YTW) was female. |
| 5 | Experience and training | *What experience or training did the researcher have?* | Methods, Data collection: Had received training in qualitative research methodology. |
| **Relationship with participants** | | | |
| 6 | Relationship established | *Was a relationship established prior to study commencement?* | Methods, Trustworthiness: YTW was a clinical nurse at the study site, holding a dual role as clinician and researcher. Insider knowledge facilitated rapport. |
| 7 | Participant knowledge of the interviewer | *What did the participants know about the researcher?* | Methods, Setting and ethics: Participants were informed of the study purpose, assured of anonymity, and told withdrawal would not affect care. |
| 8 | Interviewer characteristics | *What characteristics were reported about the interviewer/facilitator?* | Methods, Trustworthiness: Dual role as clinician-researcher; positionality documented in reflective journal. |
| ***Domain 2: Study design*** | | | |
| **Theoretical framework** | | | |
| 9 | Methodological orientation and theory | *What methodological orientation was stated?* | Methods, Study design: Descriptive qualitative study guided by the COM-B model and TDF; directed content analysis. |
| **Participant selection** | | | |
| 10 | Sampling | *How were participants selected?* | Methods, Participants and sampling: Purposive sampling using a 2×2 maximum variation matrix (CPAP adherence × receipt of structured CBT-I). |
| 11 | Method of approach | *How were participants approached?* | Methods, Participants and sampling: Recruited from inpatients at the study site between September 2025 and February 2026. |
| 12 | Sample size | *How many participants were in the study?* | Results: 16 participants (4 per group). |
| 13 | Non-participation | *How many people refused to participate or dropped out? Reasons?* | No participant withdrew or declined to complete the interview; retention was 100% and attrition was 0%. |
| **Setting** | | | |
| 14 | Setting of data collection | *Where was the data collected?* | Methods, Data collection: In a quiet room on the ward. |
| 15 | Presence of non-participants | *Was anyone else present besides the participants and researchers?* | No other persons were present during interviews (implied by “individual, face-to-face interviews in a quiet room”). |
| 16 | Description of sample | *What are the important characteristics of the sample?* | Results: Table 1 presents detailed demographics; age, sex, education, marital status, AHI, ISI, HAMA, HAMD, CPAP use, and structured CBT-I receipt. |
| **Data collection** | | | |
| 17 | Interview guide | *Were questions, prompts, guides provided by the authors?* | Methods, Data collection: Interview guide based on COM-B dimensions with four modules; provided as Additional File 2. |
| 18 | Repeat interviews | *Were repeat interviews carried out?* | No. Single interviews were conducted with each participant. |
| 19 | Audio/visual recording | *Did the research use audio or visual recording?* | Methods, Data collection: All interviews were audio-recorded with participants’ consent. |
| 20 | Field notes | *Were field notes made during and/or after the interview?* | Methods, Data collection: Field notes documenting non-verbal cues were recorded during and immediately after each interview. |
| 21 | Duration | *What was the duration of the interviews?* | Methods, Data collection: 40–60 minutes each. |
| 22 | Data saturation | *Was data saturation discussed?* | Methods, Participants and sampling: Informational saturation observed after participants 13–14; all 16 retained for matrix integrity. |
| 23 | Transcripts returned | *Were transcripts returned to participants for comment?* | Transcripts were not returned to participants. Transcripts were verified against recordings by a second researcher (TL). |
| ***Domain 3: Analysis and findings*** | | | |
| **Data analysis** | | | |
| 24 | Number of data coders | *How many data coders coded the data?* | Methods, Data analysis: Two coders. YTW coded all 16 transcripts; TL independently coded 4 transcripts for inter-rater reliability. |
| 25 | Description of the coding tree | *Did authors provide a description of the coding tree?* | Methods, Data analysis: Coding manual v2.0 with 20 nodes across 4 dimensions described. Table 2 presents theme/sub-theme structure. Figure 1 illustrates the thematic framework. |
| 26 | Derivation of themes | *Were themes identified in advance or derived from the data?* | Methods, Study design & Data analysis: Directed content analysis with COM-B/TDF as a priori framework, while remaining open to emergent themes (X3 added inductively). |
| 27 | Software | *What software was used to manage the data?* | Methods, Data analysis: NVivo 14 (Lumivero). |
| 28 | Participant checking | *Did participants provide feedback on the findings?* | Methods, Data collection: Member checking was not conducted; transcripts were verified against recordings by TL. |
| **Reporting** | | | |
| 29 | Quotations presented | *Were participant quotations presented to illustrate the themes/findings?* | Results: Verbatim quotations with participant ID, group, and demographic identifiers presented throughout all sub-themes. |
| 30 | Data and findings consistent | *Was there consistency between the data presented and the findings?* | Yes. Each sub-theme is supported by multiple quotations and reference point counts. Cross-group comparison in Results and Table 3. |
| 31 | Clarity of major themes | *Were major themes clearly presented in the findings?* | Results: Three major themes with nine sub-themes clearly delineated, with reference point counts and participant coverage reported. |
| 32 | Clarity of minor themes | *Is there a description of diverse cases or discussion of minor themes?* | Results and Discussion: Diverse cases discussed (e.g., P15 zero M7 triggers; P16 zero O2 triggers; P14 adherence illusion). X3 culturally specific beliefs discussed separately. |
