# Supplementary material for: “I know a little, but I can’t do more”: a COM-B-based qualitative study of CPAP use and sleep behaviour change among hospitalised patients with COMISA in China
Source: BMC Pulm Med. 2026 Sep 7;26:429. doi: 10.1186/s12890-026-04571-x (PMC13615572; doi:10.1186/s12890-026-04571-x)
Supplement: Supplementary file 2 [file 12890_2026_4571_MOESM2_ESM.docx]

**Additional File 2**

**Semi-structured Interview Guide**

*This interview guide was developed for individual, face-to-face semi-structured interviews with hospitalised patients meeting criteria for co-morbid insomnia and sleep apnoea (COMISA). The guide covers two target behaviours — CPAP use and sleep behaviour change — and is structured around the three COM-B dimensions (Capability, Opportunity, Motivation). Estimated duration: 30–60 minutes.*

## **Opening Statement**

*“Hello, I am [name]. Today I would like to talk with you about your experiences with snoring, insomnia, and using the breathing machine. We are mainly interested in understanding what difficulties you have encountered and what has been helpful during the process of using the breathing machine and improving your sleep. Everything you share will be used for research purposes only and will be kept strictly confidential. It will not affect your treatment in any way. If there are questions you prefer not to answer, you may skip them, and you may ask to take a break or stop at any time. I will be audio-recording our conversation. Your name will be replaced by a code number when the recording is transcribed. Is that acceptable to you?”*

## **Module A: Sleep History and Diagnostic Experience (Warm-up)**

**Q1. Can you describe what your sleep is like at the moment?**

– What time do you usually go to bed? How long does it typically take you to fall asleep? How many times do you wake up during the night?

– How long have these sleep problems lasted?

– How do these sleep problems affect your daytime life? (e.g., energy, mood, work performance)

**Q2. Looking back, how did you first discover or learn that you had a snoring problem? What was your reaction at the time?**

– Who first noticed or told you about it? (family member, doctor, or yourself)

– After learning that you had both sleep apnoea and insomnia, what were your thoughts or concerns?

## **Module B: CPAP Use Experiences (Target Behaviour 1)**

**Q3. Tell me about your current experience with the breathing machine: when do you use it, for how long, and how does it feel?**

– Over the past week or month, approximately how many nights did you use it? How many hours per night?

– When you use it, what is the most noticeable physical sensation? (e.g., stuffiness, comfort, noise, reassurance)

– Has there been a period when you particularly wanted to use it, or particularly did not want to use it?

**Q4. From your own perspective, what skills or abilities are needed to use the breathing machine well? In which areas do you feel confident, and where do you still find it difficult?**

*[COM-B: Capability]*

– For example, fitting the mask, adjusting the straps, dealing with air leaks — how comfortable are you with these?

– What explanations or demonstrations have doctors or nurses given you? How well do you feel you understood them?

– Is there anything you can understand in theory but find difficult to do in practice?

**Q5. In your hospital or home environment, what factors make it easier for you to keep using the breathing machine? And what makes it harder?**

*[COM-B: Opportunity]*

– Regarding the physical environment: bed setup, electrical outlets, machine noise, mask choice, machine maintenance — have these caused convenience or difficulty?

– What is the attitude of your family, roommates, or healthcare providers toward your use of the machine? Any encouragement, interruptions, or complaints about noise?

– Do work hours, business trips, or night shifts affect your ability or opportunity to use it?

**Q6. How do you personally feel about “wearing the breathing machine”? Under what circumstances are you more willing to wear it, and when are you less willing?**

*[COM-B: Motivation]*

– What do you see as the benefits or drawbacks of wearing the machine?

– Are there any worries, fears, or feelings of embarrassment that influence whether you wear it or not?

– Has there been a moment when you felt “this machine actually works”? Or a moment when you thought “forget it, I’m not wearing it”?

## **Module C: Insomnia Management and Sleep Behaviour Change (Target Behaviour 2)**

**Q7. During this period, what have you tried in order to sleep better? (Including methods taught by doctors or nurses, or things you found on your own)**

– For example: adjusting your sleep schedule, reducing naps, avoiding phone use before bed, relaxation exercises, medication — which of these have you tried?

– Which methods do you feel have been helpful for you?

– Are there methods you feel “make sense in theory but are hard to stick with”?

**Q8. If you were to follow the advice given by doctors or nurses — such as keeping a fixed wake-up time, limiting time in bed, or reducing screen time before sleep — what is the most difficult part for you? Do you feel you still lack certain knowledge or skills?**

*[COM-B: Capability]*

– Is there any recommendation you do not fully understand in terms of “what exactly to do”?

– When trying to follow these recommendations, have you experienced feeling “more anxious the harder I try” or “not sure if I’m doing it right”?

– Would you like someone to guide you more closely, for example helping you plan a daily routine?

**Q9. In your current living situation (family, work, night shifts, caregiving responsibilities, etc.), what factors affect whether you can follow these sleep recommendations?**

*[COM-B: Opportunity]*

– Do your family members’ or partner’s sleep schedules, snoring, or caregiving duties (for elderly parents or children) affect you?

– How do work hours, night shifts, phone use, and living conditions (light, noise) affect you?

– Is there any external support that has actually helped, such as reminders from family, fellow patients in the ward, or healthcare providers?

**Q10. How important is it to you personally to “change your lifestyle habits in order to sleep better”? What thoughts, emotions, or concerns make you more willing to try, or make you feel “forget it, it’s too much trouble”?**

*[COM-B: Motivation]*

– How much do you feel your current sleep problems affect your health, work, and relationships?

– In your daily life, what things conflict with getting good sleep? (e.g., work, leisure, family time)

– Is there something that, if provided, would make you more willing to persist?

## **Module D: Expectations of Nurse-led Support (Linking to BCW)**

**Q11. In your situation — using the breathing machine while also dealing with insomnia — how do you think nurses could help you in a way that would genuinely be useful?**

– For example: explaining the condition and treatment rationale in more detail? Teaching device operation more specifically? Helping you create a personalised sleep schedule?

– Would regular follow-up visits, phone calls, or WeChat check-ins be helpful for you?

– Which communication format do you prefer: face-to-face, telephone, group chat, printed booklets, or short videos?

**Q12. If you could design the ideal comprehensive sleep management plan for yourself (including both the breathing machine and insomnia management), what would it look like?**

– Would you prefer an intensive short-term programme or a gradual long-term adjustment?

– Would you like family members to be involved, or do you prefer working with professionals one-on-one?

– Are there any past experiences — good or bad — with healthcare that could serve as a reference?

## **Closing Statement**

*“Thank you very much for taking the time to share your experiences with me today. What you have told us is very important for improving how we help patients in the future. If you think of anything else you would like to add later, please feel free to let us know. I wish you all the best with your treatment and rest going forward.”*
