# Supplementary material for: “I know a little, but I can’t do more”: a COM-B-based qualitative study of CPAP use and sleep behaviour change among hospitalised patients with COMISA in China
Source: BMC Pulm Med. 2026 Sep 7;26:429. doi: 10.1186/s12890-026-04571-x (PMC13615572; doi:10.1186/s12890-026-04571-x)
Supplement: Supplementary file 3 [file 12890_2026_4571_MOESM3_ESM.docx]

**Additional File 3**

**Reflective Journal: Selected Excerpts**

*The first author (YTW) maintained a reflective journal throughout the coding process, comprising one overarching methodological reflection and 16 individual post-coding entries (one per participant). The following excerpts have been selected to illustrate key reflexive processes: coding manual iteration, researcher–clinician role conflict, cultural coding boundary decisions, the emergence of novel analytical concepts, and self-monitoring of coder maturation. All entries have been translated from the original Chinese by the research team.*

## Excerpt 1: Overall Methodological Reflection

*Date: 27 March 2026 | Type: Coding iteration review*

After completing the initial round of coding on the first ten transcripts, the researcher paused the coding work for several weeks to participate in another research project (a systematic review and meta-analysis). Upon returning to the data, a systematic review of the initial coding revealed several issues: some codes had remained at the parent COM-B level without sufficient differentiation into TDF sub-domains; some meaningful text segments had not been coded; the operational boundaries between certain nodes were insufficiently clear, leading to hesitation in coding decisions; and the residual category (Other) had absorbed content that would have been better captured within the COM-B framework.

Based on this review, the coding manual was revised to version 2.0. Each node was given a refined operational definition with explicit inclusion and exclusion rules and guidance for distinguishing commonly confused nodes. A new sub-node (X3: culturally specific beliefs) was added to capture phenomena specific to the Chinese cultural context that could not be fully accommodated within the standard COM-B framework. A split-coding rule was also established: when a single passage contained both objective factual content and subjective attitudinal content, it was to be coded under the respective nodes separately. All 16 transcripts were then re-coded in a new NVivo 14 project using this revised manual; the original coding was retained in the initial project as an audit trail.

## Excerpt 2: Researcher–Clinician Role Conflict (P02)

*Date: 29 March 2026 | Participant: P02, Group D, female, age 53, primary school education*

***Researcher reflection:***

A role conflict requiring transparent documentation occurred during this interview. When P02 expressed “only people who are about to die wear ventilators,” the researcher’s clinical nursing instinct took precedence, and she proactively corrected P02’s misconception about CPAP (“The breathing machine I am referring to is not the kind used for critically ill patients”). Although this intervention was clinically necessary, it constituted researcher intrusion in the context of a qualitative interview and may have influenced P02’s subsequent natural expression. In future interviews, greater vigilance is needed regarding the boundary between the researcher and clinician roles.

A second noteworthy observation concerned potential social desirability bias. P02 was emotionally intense and openly resistant during the first half of the interview (regarding CPAP), but became highly compliant during the second half (regarding CBT-I), answering almost every question with “not difficult” or “I can do it.” Given her Group D classification, low educational attainment, and the strong emotions expressed earlier, this sudden, comprehensive compliance is highly suggestive of a social desirability effect — having already refused CPAP, she may have subconsciously wished to appear more cooperative in the subsequent section. This observation serves as a reminder that when analysing CBT-I-related codes, apparent “full agreement” responses must be interpreted cautiously in light of the overall interview context.

## Excerpt 3: Cultural Coding Boundary Decision (P04)

*Date: 29 March 2026 | Participant: P04, Group B, female, age 69, junior high school, farmer, widowed*

***Coding findings (excerpt):***

X3 (culturally specific beliefs) was triggered three times in P04’s transcript, yielding the richest cultural content encountered up to this point. Three distinct cultural phenomena emerged. First, collective denial at the community level: “In our view, there is absolutely no need to go to hospital for this — this is not an illness.” The use of “our” rather than “my” indicated that this was a community-level consensus rather than an individual opinion, meeting the X3 criterion of reflecting collectively held norms rather than individual cognitions. Second, a traditional Chinese medicine attribution framework appeared for the first time: “Poor sleep leads to yin deficiency.” This represented a culturally specific causal model with no direct equivalent in the Western biomedical framework. Third, a hygiene barrier unique to the rural living environment was identified: flea infestation from free-range poultry entering the bed — an environmental obstacle that would be entirely absent from Western sleep medicine literature.

These findings, together with P02’s death-taboo associations with CPAP (“cursing oneself”) and P03’s folk belief (“snoring means sleeping soundly”), established X3 as a thematically coherent and analytically productive node. The coding boundary was clarified: X3 captures collectively held cultural norms and folk beliefs; individual misconceptions shaped by media exposure or personal experience are coded within the COM-B framework (C1 for knowledge deficits, M8 for identity-related stigma).

## Excerpt 4: Discovery of the “Adherence Illusion” (P14)

*Date: 1 April 2026 | Participant: P14, Group C, male, age 18, student, comorbid anxiety and depression*

***Coding findings (excerpt):***

P14 exhibited a behavioural pattern not seen in any of the preceding thirteen participants: an extreme split between inpatient compliance and post-discharge intention. During hospitalisation, he wore CPAP every night and cooperated fully with all treatment, even stating “I should cooperate rather than resist.” However, this compliance was not grounded in understanding or intrinsic motivation but in a role-based awareness that “being in hospital means being treated.” Simultaneously, he attributed all his problems to “anxiety and depression,” thereby fundamentally denying the value of both CBT-I and CPAP at the attribution level. His stated intention to discontinue CPAP after discharge was not a relapse but the release of a pre-existing true intention that had been masked by institutional compliance.

***Researcher reflection:***

P14’s statement “I should cooperate rather than resist” prompted the researcher to reflect on the definition of a “good patient.” In clinical practice, “compliance during hospitalisation” is typically interpreted as a positive signal. P14 demonstrated that compliance may be nothing more than surface-level role performance, with the patient’s true attitude being entirely different underneath. If the nursing team sees only his “compliance” and overlooks his “non-acceptance,” post-discharge non-adherence becomes likely. This reflection contributed to the concept of an “adherence illusion” developed in the analysis: nursing assessment should evaluate not only behavioural compliance (what the patient does) but also cognitive compliance (what the patient thinks), particularly for patients whose inpatient compliance appears externally driven.

## Excerpt 5: Coder Maturation and Reference Point Trends (P15)

*Date: 2 April 2026 | Participant: P15, Group C, male, age 60, engineer*

***Researcher reflection (excerpt):***

P15 triggered 15 nodes with 56 reference points, which was on the lower end compared with the overall study mean of approximately 73. Reviewing the trajectory from P01–P07 (mean approximately 83 reference points per transcript) to P08–P15 (mean approximately 66), a declining trend was apparent. Three possible explanations were considered: (1) a grouping effect, as later transcripts included more Group C participants whose lower barrier profiles produced more concise narratives; (2) interviewing maturation, as the researcher’s probing became more precise over time, enabling participants to reach core topics more quickly; and (3) a coder maturation effect, whereby increasing familiarity with node boundaries led to more decisive coding and potentially stricter application of inclusion criteria in later transcripts.

The third explanation warrants particular attention, as it implies that coding standards may have shifted slightly between the early and late phases. The inter-rater reliability testing was designed to detect this potential drift: the four transcripts selected for independent coding by the second coder (P08, P13, P05, P02) were sampled across the full coding timeline. If the second coder’s reference point counts for earlier transcripts (e.g., P02) also substantially exceeded those for later transcripts (e.g., P13), this would suggest the variation originates from the data itself; if the counts are comparable, it may indicate that the first author’s coding became more conservative over time. This self-monitoring process is itself part of the coder maturation trajectory and is documented here as part of the audit trail.

***Note.*** *The complete reflective journal (16 individual entries plus one overarching methodological reflection, in the original Chinese) is available from the corresponding author upon reasonable request.*
